# Sirt2 Regulates Liver Metabolism in a Sex-Specific Manner

**DOI:** 10.3390/biom14091160

**Published:** 2024-09-15

**Authors:** Alexandra V. Schmidt, Sivakama S. Bharathi, Keaton J. Solo, Joanna Bons, Jacob P. Rose, Birgit Schilling, Eric S. Goetzman

**Affiliations:** 1 Department of Pediatrics, School of Medicine, University of Pittsburgh, Pittsburgh, PA 15224, USA; avs57@case.edu (A.V.S.); bharathiss@upmc.edu (S.S.B.); kjs158@pitt.edu (K.J.S.); 2 The Buck Institute for Research on Aging, Novato, CA 94945, USA; jbons@buckinstitute.org (J.B.);

**Keywords:** sirtuin, sirt-2, liver, glucose, metabolism, gluconeogenesis

## Abstract

Sirtuin-2 (Sirt2), an NAD+-dependent lysine deacylase enzyme, has previously been implicated as a regulator of glucose metabolism, but the specific mechanisms remain poorly defined. Here, we observed that *Sirt2−/−* males, but not females, have decreased body fat, moderate hypoglycemia upon fasting, and perturbed glucose handling during exercise compared to wild type controls. Conversion of injected lactate, pyruvate, and glycerol boluses into glucose via gluconeogenesis was impaired, but only in males. Primary *Sirt2−/−* male hepatocytes exhibited reduced glycolysis and reduced mitochondrial respiration. RNAseq and proteomics were used to interrogate the mechanisms behind this liver phenotype. Loss of Sirt2 did not lead to transcriptional dysregulation, as very few genes were altered in the transcriptome. In keeping with this, there were also negligible changes to protein abundance. Site-specific quantification of the hepatic acetylome, however, showed that 13% of all detected acetylated peptides were significantly increased in *Sirt2−/−* male liver versus wild type, representing putative Sirt2 target sites. Strikingly, none of these putative target sites were hyperacetylated in *Sirt2−/−* female liver. The target sites in the male liver were distributed across mitochondria (44%), cytoplasm (32%), nucleus (8%), and other compartments (16%). Despite the high number of putative mitochondrial Sirt2 targets, Sirt2 antigen was not detected in purified wild type liver mitochondria, suggesting that Sirt2’s regulation of mitochondrial function occurs from outside the organelle. We conclude that Sirt2 regulates hepatic protein acetylation and metabolism in a sex-specific manner.

## 1. Introduction

Sirtuins play a crucial role in cellular homeostasis by regulating biochemical pathways through the reversal of lysine post-translational modifications (PTMs). Sirtuins remove acetyl, succinyl, malonyl, myristoyl, and glutaryl acyl chains, amongst others, from lysine residues of proteins and enzymes in an NAD+-dependent manner. Because NAD+ levels tend to decline with advanced age, the dependence of sirtuins on NAD+ has connected them to the pathogenesis of age-related disorders [1,2]. Among the seven sirtuins, sirtuin-2 (Sirt2) remains one of the least understood.

Sirt2 localizes to both the nucleus and cytosol. Sirt2’s association with microtubules and its role as a deacetylase of α-tubulin at lysine residue 40 (K40) underscore its involvement in microtubule stabilization and cytoskeleton remodeling [3,4,5,6]. Beyond its cytosolic functions, Sirt2 influences the cell cycle from the nucleus, impacting the G1/S and G2/M transitions and participating in genome integrity through interactions with the anaphase-promoting complex/cyclosome (APC/C) [7,8,9].

Recent studies suggest that Sirt2 may also regulate metabolism. In the context of hepatic metabolism, Sirt2 emerges as a protector against fatty liver disease and hepatic steatosis by stabilizing HNF4α via deacetylation [10], thus promoting the expression of genes involved in lipid, amino acid, and glucose metabolism, and influencing inflammatory networks [11]. Further, Sirt2 overexpression in hepatocytes has been shown to attenuate oxidative stress, support mitochondrial function, and improve insulin sensitivity [12]. In human cell lines, Sirt2 has been shown to regulate gluconeogenesis via stabilizing the rate-limiting step of gluconeogenesis, the reversible conversion of oxaloacetate into phosphoenolpyruvate (PEP) through phosphoenolpyruvate carboxykinase-1 (Pepck1) [13]. Acetyl groups accumulate on lysine residues of Pepck1 when glucose levels are high, which enhances its interactions with ubiquitin ligase to promote its degradation and the overall suppression of gluconeogenesis. Conversely, when glucose levels are low, Pepck1 is deacetylated by Sirt2, increasing Pepck1 activity, and promoting gluconeogenesis [14,15].

While some evidence supports Sirt2’s role in metabolic regulation, a gap persists in understanding its mechanistic underpinnings in a non-disease, in vivo context. This study aims to bridge this gap, specifically focusing on Sirt2’s role in hepatic metabolism in mice.

## 2. Materials and Methods

### 2.1. Animals

All animal protocols were approved by the University of Pittsburgh Institutional Animal Care and Use Committee (IACUC), and all experiments were conducted in accordance with the guidelines and regulations set forth in the Animal Welfare Act (AWA) and PHS Policy on Humane Care and Use of Laboratory Animals. All mice were maintained on a 12 h light/dark cycle in a pathogen-free barrier facility. Sirt2^Tm1.Fwa^ (*Sirt2−/−*) mice on the C57BL/6J strain were sourced from Jackson Laboratories (Bar Harbor, ME). This strain is a constitutive, whole-body knockout of Sirt2 due to the deletion of exons 5, 6, and part of 7. Mice were crossed with C57BL/6J wild type (*Sirt2+/+*) mice also sourced from Jackson Laboratories (Bar Harbor, ME, USA). Heterozygous males and females were used to generate *Sirt2+/+* and *Sirt2−/−* experimental animals.

### 2.2. Exercise Challenge

Mice ran to exhaustion on the Exer3/6 rodent treadmill (Columbus Instruments, Columbus, OH, USA). Naïve mice were acclimated to the treadmill for five minutes with the belt off and foot shock grids on and set to 40 Hz, followed by 5 min of walking (8 m/min) and five minutes of slow running at 10 m/min. Then, speed was increased at a rate of 0.5 m/min every minute up to 14 m/min and held there until mice ran a total of 500 m. Finally, mice were subjected to a second acceleration phase (0.5 m/min increase per minute) until exhaustion. Exhaustion was determined by mice sitting on a foot shock grid for at least 5 s, then removed from the apparatus. Blood glucose and lactate levels were measured with hand-held meters from Germaine Laboratories (San Antonio, TX, USA) and EKF Diagnostics (Cardiff, Wales, UK), respectively.

### 2.3. Indirect Calorimetry

Indirect calorimetry (IDC) was performed using the Comprehensive Lab Animal Monitoring System (CLAMS, Columbus Instruments, Columbus, OH, USA). Mice were acclimated to the special caging for 24–48 h prior to a 72 h continuous monitoring period. Pelleted food was placed in the cages and available to mice throughout the duration of the experiment. Measurements were normalized to total body mass. Body composition was determined using the EchoMRI-100 system (Echo Medical Systems, Houston, TX, USA).

### 2.4. Immunoblotting

Tissue lysates in buffer containing 0.05% NP-40, 50 mM NaCl, 0.5 mM EDTA, 50 mM Tris-HCl, and 10 mM NAM with 1× EDTA-free protease inhibitor (Roche, St. Louis, MO, USA) were electrophoresed on Criterion SDS polyacrylamide gels (BioRad, Hercules, CA, USA) and transferred to nitrocellulose membranes. Primary antibodies used were anti-AcK (Cell Signaling, Danvers, MA, USA), anti-Sirt2 (Abcam, Cambridge, UK), anti-actin (Protein Tech, Rosemont, IL, USA), anti-alpha-tubulin (Protein Tech), anti-Lamin A/C (Protein Tech), anti-ACOX1 (Thermo Fisher, Waltham, MA, USA), and anti-TIMM23 (Thermo Fisher). After incubation with 1: 10,000 HRP-conjugated secondary Goat anti-Rabbit-HRP IgG or Goat anti-Mouse-HRP IgG antibody (Bio-Rad, Hercules, CA, USA) blots were visualized with Clarity Max ECL (Bio-Rad, Hercules, CA, USA) and in some cases, scanned and subjected to densitometric analysis using ImageJ software version 1.52o (imagej.nih.gov).

### 2.5. Quantitative Mass Spectrometry

Liver samples were snap-frozen and stored at −80 °C until the time of analysis (*N* = 4 for male mice, *N* = 3 for female mice). Tissue pieces were homogenized, trypsinized, and desalted. For every sample, one aliquot was used for whole lysate (protein level) analysis and the other aliquot was used to enrich for acetylated peptides using the PTMScan Acetyl-Lysine Motif Kit (Cell Signaling Technologies, Danvers, MA, USA) as previously described. Using reversed-phase high-performance liquid chromatography/electrospray ionization–tandem mass spectrometry, acetyl-enriched samples were analyzed by data-independent acquisition (DIA) [16,17,18] on an Orbitrap Eclipse mass spectrometer and site-specific changes in acetylation were quantified using library-free directDIA in Spectronaut (Biognosys, Schlieren, Switzerland) [19,20] (see Supplemental Tables S1 and S2). Matching whole lysate liquid chromatography with tandem mass spectrometry analysis conducted on the same system operated in DIA-MS was used to determine total protein abundance changes (see Supplemental Tables S4 and S5), and to normalize PTM site changes adjusting for the protein-level changes. Pathway analysis was conducted using the Reactome Analysis Tools online accessed on 15 April 2022 (reactome.org/PathwayBrowser).

### 2.6. Co-Immunoprecipitation

Freshly collected *Sirt2−/−* liver was homogenized in low-stringency NP1 buffer (1% nonident P-40, 300 mM NaCl, 50 mM Tris-HCl) with EDTA-free Protease Inhibitor Cocktail (Roche, St. Louis, MO, USA), nicotinamide (NAM) (Sigma-Aldrich Co., St. Louis, MO, USA), and Trichostatin A (TSA) (Sigma-Aldrich). A total of 1 mg of liver homogenate was pre-cleared with anti-His6 (1:500, Roche, St. Louis, MO, USA) then incubated with 20 µg of recombinant His6-tagged human SIRT2 protein (rSIRT2-His6) (R&D Systems, Minneapolis, MN, USA) at 37 °C for 30 min in a microfuge rotator. Anti-His6 (Sigma-Aldrich) was conjugated to 20 µg of Protein A/G agarose beads (Pierce, Appleton, WI, USA) overnight. Anti-His6-A/G was added to the liver homogenate treated with rSIRT2-His6 (1:100) overnight at 4 °C. Agarose beads were pelleted, washed 3 times with NP1 buffer, and resuspended in 50 µL of 2× Laemmli buffer (Bio-Rad, Hercules, CA, USA) containing β-mercaptoethanol (EM Sciences, Hatfield, PA, USA). Samples were boiled at 80 °C for 15 min and electrophoresed on a Criterion SDS polyacrylamide gel (Bio-Rad) for ~10 min at 150 V. The gel was stained with Coomassie blue (Bio-Rad) and then bands were isolated from the gel and stored in 1 mL of ultra-pure H_2_O at 4 °C. Gel bands were processed at the University of Pittsburgh Health Sciences Mass Spectrometry Core using in-gel digestion, sample concentration, and purification with reverse-phase liquid chromatography (RP-LC). Following mass spectrometry peptides were aligned to a UniProt Mouse database (UniProt, 20190131) for identification. The average value of % coverage was calculated for each identified protein.

### 2.7. Liver Organelle Purification

Approximately 0.5 g liver tissue was homogenized using a rotor-driven homogenizer at a slow speed of ~500 rpm in a homogenization buffer containing 0.25 M sucrose and 5 mM MOPS, pH 7.2 with 0.2 mM PMFS dissolved in 0.2 mL ethanol (1% *v*/*v* final) and 1× EDTA-free Protease Inhibitor Cocktail (Roche, St. Louis, MO, USA) added fresh the day of isolation. Debris was pelleted with a 10 min 100× *g*, 4 °C spin. The supernatant was centrifuged at 600× *g* for 10 min, 4 °C to isolate the Nuclear Pellet. The supernatant was centrifuged at 1950× *g* for 10 min, 4 °C to isolate the Heavy Mitochondrial Pellet. The supernatant was centrifuged at 3000× *g* for 10 min, 4 °C to isolate the Light Mitochondrial Pellet. A total of 4 mL of the supernatant was transferred to *g* maxi RCF tubes (Thermo Fisher Scientific, Waltham, MA, USA) containing 2 mL of 27.5% Optiprep Density Gradient Medium (Sigma, St. Louis, MO, USA) carefully, laying on top of the denser 2 mL fraction. A total of 2 mL of 20% Optiprep Density Gradient Medium (Sigma, St. Louis, MO, USA) was added carefully to the top of the supernatant to create a sucrose density column. Samples were spun overnight for ~15 h at 10,000× *g*, 4 °C. Mitochondria and Peroxisome pellets were collected and lysed immediately for immunoblotting.

### 2.8. Triglyceride Content Assay

A total of 100 mg of snap-frozen liver tissue was added to 350 µL ethanolic KOH (2 parts 100% EtOH; 1 part 30% KOH) and incubated at 55 °C overnight. The sample volume was brought to 1 mL 44 with 50% EtOH (about 700 µL) and spun using a tabletop microcentrifuge. The supernatant was added to a new tube and the volume was brought to 1.2 mL with 50% EtOH (about 300 µL). A total of 200 µL of samples were added to 215 µL of MgCl_2_. Glycerol content was assayed using a plate spectrometer at 540 nm compared to a glycerol standard (0–2.5 mg/mL; Sigma-Aldrich Co., St. Louis, MO, USA). Sample concentrations were obtained from a linear regression fit and liver TG content was calculated using the following formula, where TGconc = triglyceride concentration; TG content = x × (415/200) × 0.012 (dL/mg tissue) [21].

### 2.9. Lactate, Pyruvate, and Glycerol Tolerance Tests

To reduce stress response to needles, all mice were handled and injected with 10 µL of sterile 1x PBS per g body weight twice prior to the day of the experiment. For lactate tolerance testing, mice received a 2 g/kg intraperitoneal (i.p.) injection of 200 g/kg sodium L-lactate, pH 7.4 (Sigma-Aldrich Co., St. Louis, MO, USA) dissolved in sterile 1x PBS; corresponding to 18 mmol/kg. Blood glucose and lactate levels were measured with hand-held meters from Germaine Laboratories (San Antonio, USA) and EKF Diagnostics (Cardiff, Wales, UK), respectively, prior to injection (baseline) then 5, 10, 15, and 30 min post-injection [22]. For pyruvate tolerance testing, mice were fasted for 12 h prior to the experiment to deplete glycogen stores. Mice received a 10 µL/1 g of body weight i.p. injection of 0.3 g/mL sodium pyruvate, pH 7.4 (Sigma-Aldrich Co., St. Louis, MO, USA) dissolved in sterile 1× PBS. Blood glucose and lactate levels were measured with hand-held meters prior to injection (baseline) then 10, 20, 30, 45, 60, 90, and 120 min post-injection [23]. For glycerol tolerance testing, 6-h fasted mice were injected i.p. with glycerol at 1 mg per g of body weight. Blood glucose and lactate levels were measured with hand-held meters prior to injection (baseline) and then 15, 30, 60, 90, and 120 min post-injection.

### 2.10. LDH Activity Assay

A total of 100 mg of liver tissue lysate was prepared in 500 µL of LDH Assay Buffer (Sigma-Aldrich Co., St. Louis, MO, USA) containing 1× EDTA-free protease inhibitors (Sigma-Aldrich Co., St. Louis, MO, USA) and centrifuged for 15 min at 10,000× *g*, 4 °C. Samples were diluted 1:25 in LDH Assay Buffer containing 1× protease inhibitors (Roche, St. Louis, MO, USA), followed by another 1:25 dilution in LDH Assay Buffer containing 1× Protease Inhibitor Cocktail (Roche, St. Louis, MO, USA), resulting in a 1:625 working dilution. A total of 50 µL of dilute samples were added in triplicate to a 96-well plate. NADH standards were prepared according to the Lactate Dehydrogenase Activity Assay Kit (Sigma-Aldrich Co., St. Louis, MO, USA) protocol. The plate was shaken for 3 min at RT to mix reagents, protected from light and then removed for T_initial_ reading at 450 nm. The plate was then incubated in a non-CO_2_ 37 °C incubator and read every 5 min until the sample absorbance reading exceeded the highest standard (12.5 mol/well) reading, with the penultimate reading defined as T_final_. Sample NADH concentrations were linear regression and LDH activity was calculated with the equation B((15 − 3) × 0.05) × 1000 where B = amount (nmol) of NADH generated between T_initial_ (3 min) and T_final_ (15 min).

### 2.11. High-Resolution Respirometry

Freshly isolated wild-type and *Sirt2−/−* mouse liver mitochondria were assessed using an Oroboros Oxygraph-2K (Oroboros, Österreich, Austria) [24]. The 100 mg of fresh liver was isolated, minced and then placed into 500 µL of isolation buffer containing 225 mM mannitol, 75 mM sucrose, 10 mM Tris pH 7.4, and 0.2 mM EDTA. Tissues were homogenized by mechanical shearing and mitochondria were isolated after one 1000× *g* and another 6200× *g* spin. Intact mitochondria were resuspended in 100 µL of isolation buffer. A total of 60 µL of mitochondria (approximately 30 µg) were added to O_2_-equilibrated chambers containing 5 mL isolation buffer. After the baseline became stable, 10 μm cytochrome C (Sigma, St. Louis, MO, USA) was added to assess mitochondrial integrity. Then, malate (5 mm, Sigma, St. Louis, MO, USA), ADP (2 mm, Sigma-Aldrich Co., St. Louis, MO, USA), pyruvate (5 mm, Sigma-Aldrich Co.), glutamate (5 mM, Sigma-Aldrich Co.), and succinate (5 mM, Sigma-Aldrich Co.) were added to stimulate State 3 respiration. A total of 2 µL carbonyl cyanide m-chlorophenyl hydrazone (CCCP, Sigma-Aldrich Co.) was added to uncouple the ETC, then finally, 1 µL ROT/AA (Sigma, St. Louis, MO, USA) was added to stop mitochondrial respiration altogether.

### 2.12. Fatty Acid Oxidation (FAO) Flux Assays

A 1.0 × 10^6^ primary hepatocytes were placed in 1 mL incomplete 5 mM glucose DMEM (Invitrogen, Waltham, MA, USA) to mimic starvation conditions prior to assay [25]. The ^14^C-labeled palmitate and ^14^C-glucose were sourced from PerkinElmer (Waltham, MA, USA). Just prior to assay, the fatty acids were dried to completion under nitrogen and then solubilized in 10 mg/mL α-cyclodextrin via incubation at 37 °C for 30 min. FAO reactions consisted of 1.0 × 10^5^ primary hepatocytes in a total reaction volume of 100 μL containing 5 mM glucose DMEM and 125 μM of fatty acid or glucose (0.5 μCi/mL). After 1 h incubation at 37 °C, FAO reactions were stopped by adding perchloric acid to 0.5 M final concentration. Following centrifugation to remove precipitated material, the water-soluble ^14^C-labeled FAO products were isolated by methanol-chloroform extraction and quantified on a scintillation counter. The results were normalized to protein concentration.

## 3. Results

### 3.1. Deletion of Sirt2 Alters Liver Protein Acetylation in Male but Not Female Mice

**Figure 1:**
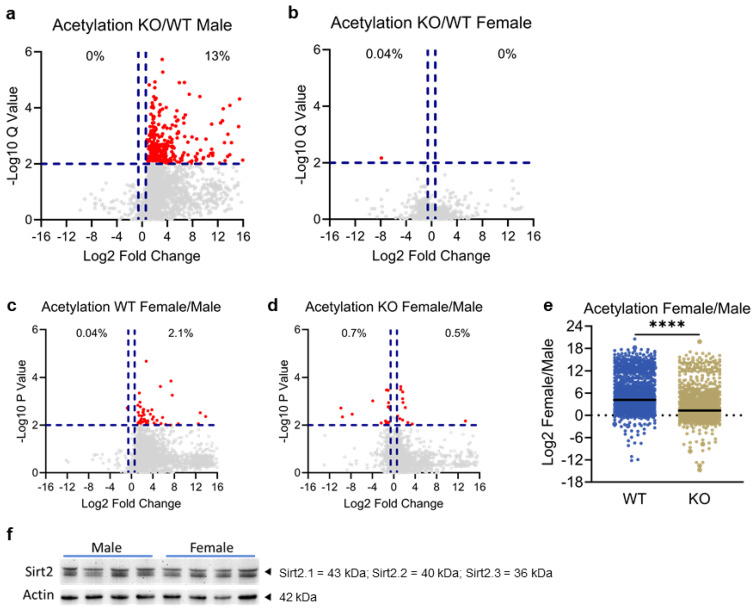
(**a**) Mass spectrometry was used to quantify acetylated peptides from male *Sirt2+/+* (WT) and *Sirt2−/−* (KO) livers. Peptides with significant changes in acetylation are rendered in red. Blue lines indicate the mean log2 fold-change. A total of 2452 acetylated peptides were quantified. The mean acetylation level significantly increased in 317 peptides (*p* < 0.01, FC > 1.5) due to Sirt2 ablation. (**b**) Mass spectrometry was used to quantify acetylated peptides from female *Sirt2+/+* and *Sirt2−/−* livers. There was no appreciable change in acetylation levels across putative Sirt2-targets. (**c**) Volcano plot of *Sirt2+/+* female to male ratios at each of the 2452 putative Sirt2 targets, expressed as Log2 FC. (**d**) Volcano plot of *Sirt2−/−* female to male ratios at each of the 2452 putative Sirt2 targets, expressed as Log2 FC. (**e**) The mean Log2 FC in *Sirt2+/+* female/male acetylation was 4.2; the mean Log2 FC in *Sirt2−/−* female/male acetylation was 1.3. Quantification and comparison of the Log2 FC ratios using a Student’s two-sided *t*-test showed that *Sirt2−/−* males had significant decrease in hyperacetylation in 2452 putative sites compared to wild type counterparts (*p* < 0.0001). (**f**) Immunoblotting for Sirt2 in livers from wild type mice indicated no sex difference in the expression of the Sirt2 protein. Note that there are multiple bands due to multiple isoforms of Sirt2. **** *p* < 0.0001 by Student’s two-sided *t*-test.

Sirt2 functions as a lysine deacetylase by removing acetyl groups from the lysine residues of proteins and enzymes, but little is known about the identity of its targets. We therefore used quantitative mass spectrometry to objectively profile the liver acetylome. Both sexes of mice (N = 3 or 4) were subjected to an overnight fast prior to liver harvest. A total of 2452 acetylated peptides were quantified. In male mice, ablation of Sirt2 increased the mean acetylation level by 8-fold (average log2 fold-change = 3 in Figure 1a), with 317 acetylated peptides, corresponding to 306 unique acetylation sites, (13%) showing a statistically significant change (*p* < 0.01, FC > 1.5) (Supplemental Table S1). In contrast, female *Sirt2−/−* mice did not show any appreciable change in hepatic protein acetylation (Figure 1b). There were no putative Sirt2 targeted lysine residues in the female liver, i.e., no sites showing significantly increased acetylation upon Sirt2 ablation. Only one acetylated peptide showed a significant change, and it was reduced rather than increased (indicated in red in Figure 1b). Interestingly, in wild type mice, a volcano plot of the female/male ratio for peptide acetylation revealed a strong trend for higher lysine acetylation in females for nearly all the 2452 measured peptides (Figure 1c), with an average log2 fold increase of 4.2. This sex effect was dampened in *Sirt2−/−* female mice, where the log2 fold increase in the liver was only 1.3 (Figure 1d,e). Of note, the higher lysine acetylation in wild type female liver was not due to lower expression of *Sirt2*, as the total amount of Sirt2 antigen did not differ between the sexes (Figure 1f).

### 3.2. Loss of Sirt2 Increases Acetylation in Multiple Cellular Compartments in the Male Liver

The 306 putative Sirt2 target sites identified in male liver mapped to 231 distinct proteins, which were subjected to pathway analysis (Reactome). The 15 pathways in which protein acetylation was most altered in *Sirt2−/−* livers were related to cellular metabolism and protein synthesis (Table 1).

**Figure 2:**
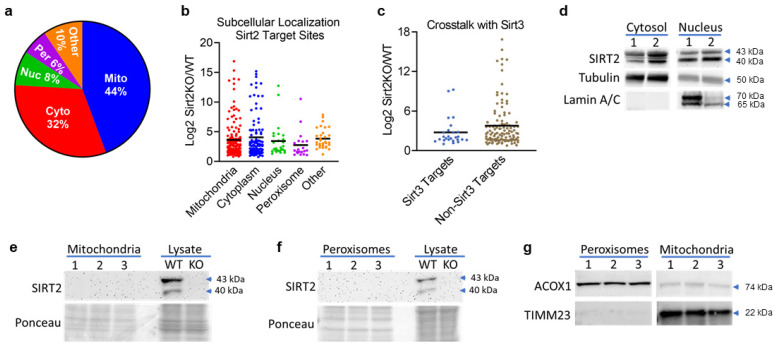
(**a**) The 306 putative Sirt2 target sites mapped to proteins spread across multiple cellular compartments but were most abundant in the mitochondria and cytosol. (**b**) A plot of the Log2 ratio (FC > 1.5) of *Sirt2−/−* vs. *Sirt2+/+* by cellular compartment revealed that the mitochondria and cytosol had the largest FCs in lysine acetylation. (**c**) The Log2 Ratio of putative mitochondrial Sirt2 targets from this study was compared to known Sirt3 targets within the mitochondria. Of the 140 Sirt2 targets represented in this plot, 19% are also Sirt3 targets. (**d**) Preparations of *Sirt2+/+* nuclear and cytosolic fractions show that Sirt2 is present in both compartments, confirmed with the cytosolic marker α-tubulin and the nuclear marker, Lamin A/C (1 and 2 denote matched, replicate samples). Sirt2 is present in both compartments, confirming acetylomics data. (**e**) Sirt2 was not detected in mitochondria prepared from *Sirt2+/+* hepatic tissue lysates (N = 3). 1–3 denotes replicate samples. (**f**) Sirt2 was not detected in peroxisomes prepared from *Sirt2+/+* hepatic tissue lysates (N = 3). 1–3 denotes replicate samples. (**g**) The ultrapure subcellular fractionation technique used completely separated mitochondria and peroxisomes, confirmed with the peroxisomal marker, ACOX1, and the mitochondrial marker, TIMM23 (N = 3). 1–3 denotes matched, replicate samples. Membranes exposed at the same time, cropped for clarity.

Sirt2 is known to localize to the nucleus and cytosol [26]. Here, we found that the 306 putative Sirt2 target sites were spread over proteins that localize to many cellular compartments, including mitochondria (44%), cytosol (32%), nucleus (8%), and peroxisomes (6%) (Figure 2a). Additional minor compartments included the endoplasmic reticulum (ER), plasma membrane, lipid droplets, and lysosomes (combined into “other” in Figure 2a). We then compared the log2 fold-change for peptide acetylation in each compartment and found that the cellular compartments with the most dynamic changes upon Sirt2 ablation were the mitochondria and cytosol (Figure 2b).

Since Sirt3, another sirtuin family member, is thought to be the major mitochondrial lysine deacetylase, we compared putative Sirt2 mitochondrial target sites with the known Sirt3 target sites delineated by Hirschey et. al. [27] to evaluate potential crosstalk between the two sirtuins. Of the 140 putative Sirt2 mitochondrial target sites, only 19% were also Sirt3 target sites, indicating only modest overlap between the two sirtuins. The subgroup of putative Sirt2 targeted peptides that are also known to be Sirt3 targets tended to have lower fold-change increases in *Sirt2−/−* liver compared to the subgroup of putative Sirt2-targeted peptides that are not Sirt3 target sites (Figure 2c), possibly due to Sirt3-mediated deacetylation of the shared target sites. Overall, the large number of putative Sirt2 mitochondrial target peptides implied a potential direct role for Sirt2 inside the mitochondria.

To further investigate this, we performed careful subcellular fractionation of wild type mouse liver. Consistent with the literature, two major Sirt2 isoforms were detected by immunoblotting of nuclear and cytosol preparations (Figure 2d). The 43 kDa isoform is believed to be primarily cytosolic, and the 39 kDa shuttles between the cytoplasm and nucleus. Density centrifugation was used to purify mitochondria from wild type liver. As part of this fractionation protocol peroxisomes were also obtained. Neither purified mitochondria nor peroxisomes were observed to contain Sirt2 antigen (Figure 2e,f), even upon extended exposure times (not shown). The fractions were confirmed to be purified peroxisomes and mitochondria via blotting for the peroxisomal marker protein acyl-CoA oxidase-1 (ACOX1) and the mitochondrial marker TIMM23 (Figure 2g). We conclude that either Sirt2 targeting of mitochondrial and peroxisomal proteins occurs prior to import into their respective organelles, or changes in acetylation in these compartments upon Sirt2 ablation is an indirect consequence of altering a cytosolic and/or nuclear pathway.

### 3.3. Sirt2 Is Not Involved in Maintenance of Global Hepatic Gene Expression

Only 8% of significantly hyperacetylated sites in *Sirt2−/−* liver belonged to nuclear proteins. Sites belonging to three key regulators of gene expression were among the most hyperacetylated: Hint1, c-Myc, and Rela (NF-ĸB) (Table 2). Additionally, we found that several histone proteins were modestly hyperacetylated in the absence of Sirt2 (Supplemental Table S1). Therefore, we used both proteomic and RNA-seq-based approaches to look for changes in protein abundance and gene expression. Surprisingly, RNA-seq showed almost no changes in the transcriptome between *Sirt2−/−* and *Sirt2+/+* livers, with only eight significantly differentially expressed genes (adjusted *p* < 0.05; Supplemental Table S3). At the protein level, 20 proteins significantly changed in abundance between *Sirt2−/−* males and *Sirt2+/+* males, and 38 between *Sirt2−/−* females and *Sirt2+/+* females (*p* < 0.01, >1.5 FC; Supplemental Tables S4 and S5). Altogether, deletion of Sirt2 had very little effect on either the transcriptome or the proteome.

### 3.4. Loss of Sirt2 Disrupts Glucose Homeostasis in Male Mice

**Figure 3:**
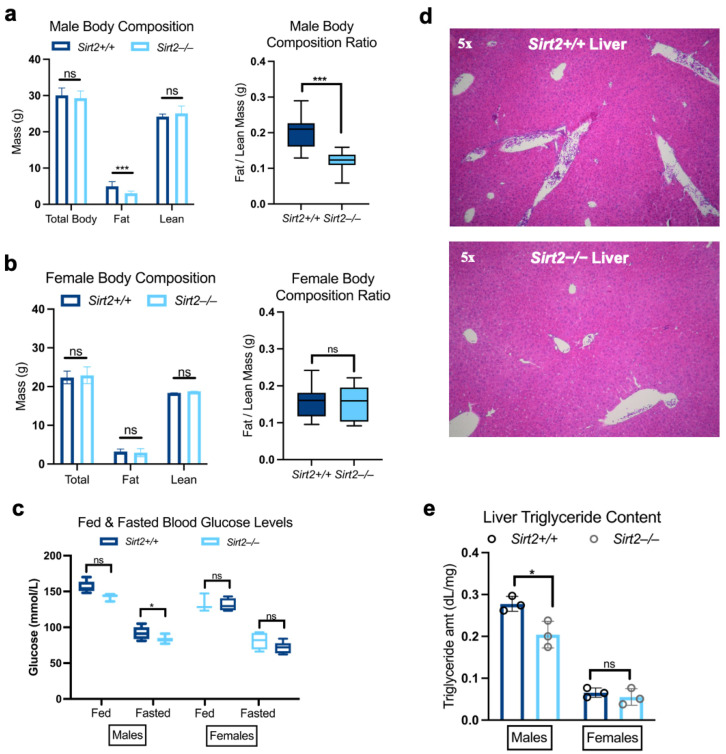
(**a**,**b**) Total body weight and lean body mass were the same between *Sirt2−/−* (N = 11) and *Sirt2+/+* (N = 8) males, but *Sirt2−/−* males had significantly lower fat mass compared to *Sirt2+/+* males (*p* < 0.05). (**b**) Total body weight, fat mass, and lean mass were not significantly different between *Sirt2−/−* (N = 6) and *Sirt2+/+* females (N = 8). Body composition was calculated for each mouse by dividing fat mass by lean mass. Only *Sirt2−/−* males had a significantly reduced fat/lean mass body composition (*p* < 0.001) compared to controls; female *Sirt2−/−* did not. (**c**) Blood glucose levels in *Sirt2−/−* vs. *Sirt2+/+* males or females in both a fed and fasted state. In a fed state, there was no difference in blood glucose levels between *Sirt2−/−* and *Sirt2+/+* in either males (N = 8) or females (N = 8). Upon fasting, only *Sirt2−/−* males had significantly reduced blood glucose levels (N = 8; *p* < 0.05) while *Sirt2−/−* females showed no significant reduction compared to controls (N = 8). (**d**) H&E staining of *Sirt2−/−* vs. *Sirt2+/+* livers indicated no obvious morphological changes or tissue damage (N = 4). (**e**) Male *Sirt2−/−* mice (N = 3) had significantly less triglycerides in hepatic tissue compared to *Sirt2+/+* males (N = 3; *p* < 0.05). Comparisons made with a Student’s nonparametric *t*-test; * *p* < 0.05, *** *p* < 0.001.

Hyperacetylated proteins in male *Sirt2−/−* liver tended to cluster in metabolic pathways (Table 1). Whether a direct or indirect effect, many mitochondrial enzymes were hyperacetylated in the absence of Sirt2, suggesting that knocking out Sirt2 perturbs hepatic metabolism. To that end, we performed basic metabolic phenotyping in *Sirt2−/−* mice. First, we observed that there was no effect on whole-body respiration, and no change in the respiratory exchange ratio (RER) (Figure S1). *Sirt2−/−* male mice had less adipose tissue compared to *Sirt2+/+* male mice but maintained a similar amount of lean mass (Figure 3a). This difference in body composition was not observed in female *Sirt2−/−* mice (Figure 3b). Upon fasting, *Sirt2−/−* males became mildly hypoglycemic, but female *Sirt2−/−* mice did not (Figure 3c). While liver histology revealed *Sirt2−/−* hepatic tissue to be overall normal (Figure 3d), male *Sirt2−/−* had significantly fewer liver fat stores than *Sirt2+/+* males (Figure 3e). Again, this difference was not seen in *Sirt2−/−* females.

**Figure 4:**
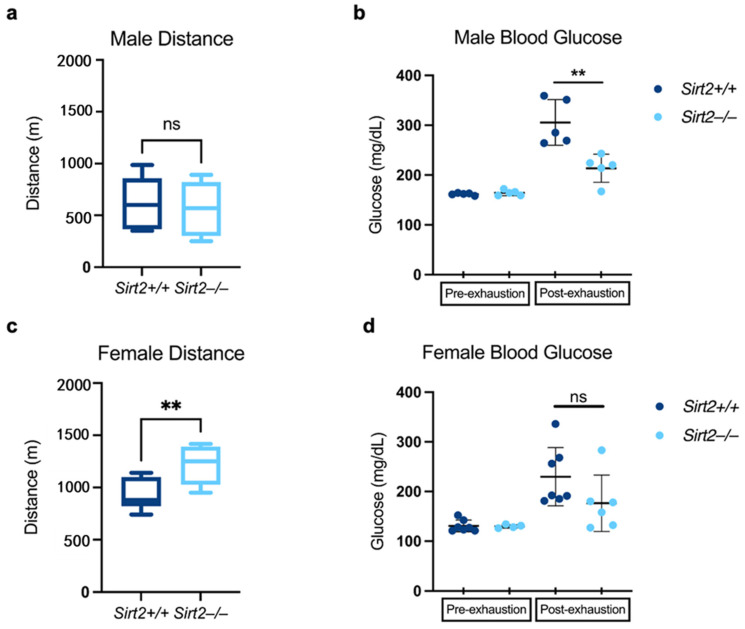
(**a**) *Sirt2−/−* and *Sirt2+/+* males ran the same distance (N = 5) before reaching exhaustion. (**b**) Blood glucose levels were normal prior to the treadmill assay in male *Sirt2−/−* and *Sirt2+/+* mice. Upon exhaustion, male *Sirt2−/−* mice (N = 5) had significantly lower blood glucose levels than *Sirt2+/+* counterparts (N = 5; *p* < 0.01). (**c**) *Sirt2−/−* females (N = 6) ran significantly further than *Sirt2+/+* females (N = 7; *p* < 0.005) before reaching exhaustion. (**d**) There was no significant difference in blood glucose levels between *Sirt2−/−* (N = 6) and *Sirt2+/+* females (N = 7) post-exhaustion. Comparisons were made using a Student’s nonparametric *t*-test; ns = no difference; ** *p* < 0.01.

Finally, we subjected the mice to a treadmill challenge in which exercise naïve mice were run to exhaustion. Male *Sirt2−/−* mice ran a similar total distance compared to their *Sirt2+/+* counterparts. Interestingly, females overall ran ~2X further than male mice, and in particular, female *Sirt2−/−* mice ran the furthest (Figure 4a,c). In *Sirt2+/+* mice of both sexes, running-induced a gluconeogenic response, such that blood glucose was significantly higher at exhaustion than it was at the start of the run (Figure 4b,d). In male *Sirt2−/−* mice, this gluconeogenic response was blunted (Figure 4b). In female *Sirt2−/−* mice, there was a similar trend, but statistically nonsignificant (Figure 4d).

**Figure 5:**
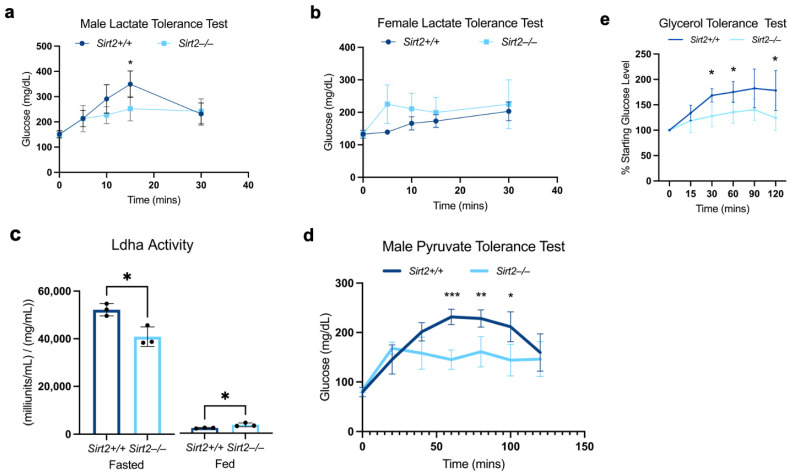
(**a**) Male *Sirt2−/−* mice had a blunted response to a bolus lactate injection, with significantly decreased glucose production 15-min post-injection (N = 5; *p* < 0.05). (**b**) *Sirt2−/−* females had a non-significantly different response to bolus lactate injection compared to *Sirt2+/+* females (N = 5). (**c**) Ldha activity in *Sirt2−/−* fasted males (N = 3) was significantly reduced compared to *Sirt2+/+* male mice (N = 3; *p* < 0.05). Ldha activity in fed *Sirt2−/−* mice was significantly higher than in *Sirt2+/+* male mice (N = 3; *p* < 0.05). (**d**) Male *Sirt2−/−* mice (N = 5) had a significantly reduced response to a pyruvate tolerance test than male *Sirt2+/+* mice (N=) 75 (*p* < 0.0005), 85 (*p* < 0.005), and 100 min (*p* < 0.05) post-injection. (**e**) *Sirt2−/−* males (N = 5) had significantly less glucose production at 30-, 60-, and 120-min post-glycerol injection (*p* < 0.05). All comparisons were made at each time point using a Student’s parametric *t*-test; * *p* < 0.05, ** *p* < 0.001, *** *p* < 0.0001.

**Figure 6:**
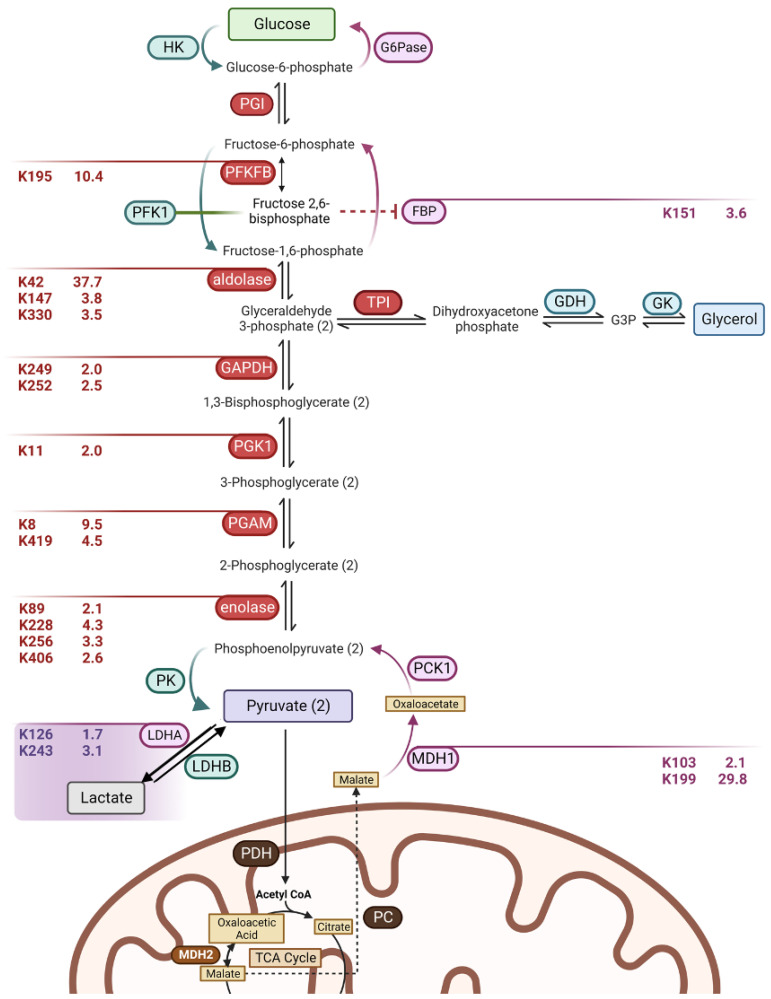
Schematic of glycolytic enzymes (red) and gluconeogenic enzymes (purple) that contain putative Sirt2 targets. Each significantly altered residue (*p* < 0.01; FC > 1.5) is identified with FC reported. Non-targets are in blue.

The relative hypoglycemia seen in male *Sirt2−/−* mice in either a fasted state (Figure 3c) or after an exercise challenge (Figure 4b) suggested a potential disruption of gluconeogenesis in male *Sirt2−/−* mice. During both fasting and exercise, the Cori cycle converts lactate released by peripheral tissues such as muscle into glucose via hepatic gluconeogenesis [28]. We tested the capacity of the Cori cycle in *Sirt2−/−* mice using lactate tolerance testing in which the animals were injected with lactate and subsequent effects on blood glucose were monitored over time. Indeed, *Sirt2−/−* males were deficient in gluconeogenesis induced during the lactate tolerance test (Figure 5a) while female *Sirt2−/−* mice did not show this defect (Figure 5b). The first step of hepatic gluconeogenesis from lactate is mediated by lactate dehydrogenase (Ldh), for which there are two isoforms (Ldha, Ldhb) with differing biochemical affinities for lactate [29]. Our proteomic analysis showed that neither Ldha nor the lesser expressed Ldhb isoform were differentially expressed in male *Sirt2−/−* livers (Supplemental Table S4). Ldha did, however, exhibit significant hyperacetylation, as did many other enzymes within the glycolytic/gluconeogenic pathway (Figure 6). Ldha activity has been shown to be suppressed by lysine acetylation [30]. In support of this, we found that Ldha activity in liver lysates from fasted *Sirt2−/−* males was significantly reduced (Figure 5c). Interestingly, Ldha activity in liver lysates prepared from fed *Sirt2−/−* mice was slightly increased compared to *Sirt2+/+* liver lysates.

We next asked whether the reduced Ldha activity in the *Sirt2−/−* male liver was sufficient to explain the blunted gluconeogenesis observed in the lactate tolerance test. To answer this, we employed pyruvate tolerance testing, reasoning that pyruvate would bypass any acetylation-associated defect in Ldha activity (see Figure 6 for an overview of the gluconeogenesis pathway). Male *Sirt2−/−* mice generated less glucose following an injection of pyruvate, indicating that the reduction in Ldha activity is not the sole mechanism by which Sirt2 ablation dampens gluconeogenesis (Figure 5d). Furthermore, gluconeogenesis was also blunted in *Sirt2−/−* males when glycerol was acutely delivered as a substrate, which distally enters the gluconeogenic pathway via triphosphate isomerase (Tpi) (Figure 5e).

### 3.5. Sirt2−/− Males Exhibit Reduced Mitochondrial Function in the Liver

**Figure 7:**
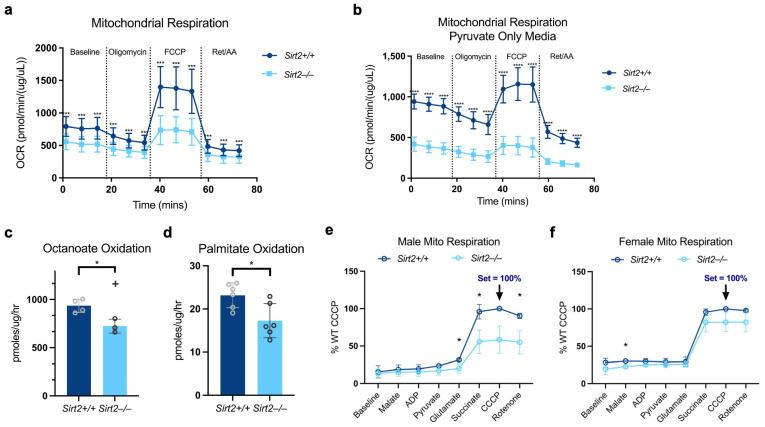
(**a**) Hepatocytes isolated from a *Sirt2−/−* liver showed reduced OCR compared to *Sirt2+/+* hepatocytes during a mitochondrial stress test using a Seahorse Extracellular Flux Analyzer (*p* < 0.005). (**b**) Hepatocytes isolated from *Sirt2−/−* liver showed significantly reduced OCR compared to *Sirt2+/+* isolated hepatocytes when given media containing only pyruvate as an energy source (*p* < 0.00001). (**c**) *Sirt2−/−* liver tissue lysates (N = 4) had a significantly reduced FAO response to palmitate (16C chain fatty acid) compared to *Sirt2+/+* liver lysate (N = 4) during a radio-labeled redox assay (*p* < 0.05). (**d**) *Sirt2-/-* liver lysates (N = 6) had a significantly reduced response to octanoate (8C chain fatty acid) compared to liver lysate prepared from *Sirt2+/+* mice (N = 6; *p* < 0.05). (**e**) The maximal mitochondrial capacity was significantly lower in mitochondria isolated from male *Sirt2−/−* mouse hepatocytes (N = 3) compared to *Sirt2+/+* isolated mitochondria (N = 3; *p* < 0.05). (**f**) The maximal mitochondrial capacity was relatively the same in mitochondria isolated from female *Sirt2−/−* mouse hepatocytes (N = 3) compared to *Sirt2+/+* isolated mitochondria (N = 3; *p* > 0.05). All comparisons were performed with a Student’s nonparametric *t*-test; * *p* < 0.05, *** *p* < 0.0001, **** *p* < 0.00001.

Gluconeogenesis requires a steady supply of ATP, typically supplied by fatty acid oxidation in the mitochondria. One explanation for the reduced gluconeogenesis in *Sirt2−/−* males on diverse substrates (lactate, pyruvate, glycerol) could be impaired ATP generation. To address this question, primary hepatocytes were isolated for Seahorse extracellular flux analysis. Male *Sirt2−/−* hepatocytes exhibited a reduced oxygen consumption rate (OCR) in complete media containing glucose, pyruvate, and glutamine, as well as in pyruvate-only media (Figure 7a,b). To measure FAO flux, male *Sirt2−/−* primary hepatocytes were also probed with ^14^C-fatty acids. FAO was suppressed in *Sirt2−/−* hepatocytes with both the medium-chain fatty acid ^14^C-octanoate and the long-chain fatty acid ^14^C-palmitate (Figure 7c,d). To probe the function of isolated mitochondria, differential centrifugation was used to prepare an enriched sample of liver mitochondria for high-resolution respirometry. This technique confirmed that indeed, male *Sirt2−/−* mitochondria were dysfunctional compared to male *Sirt2+/+* mitochondria, yet female *Sirt2−/−* mitochondria were equivalent to female *Sirt2+/+* mitochondria (Figure 7e,f).

## 4. Discussion

Here, we report the first unbiased mass spectrometry characterization of the mouse lysine liver acetylome in the context of Sirt2 ablation. These data uncovered a sexually dimorphic Sirt2-associated acetylome. This dataset also confirmed many Sirt2 targets previously reported in vitro. We found that Sirt2-mediated acetylation predominantly affected metabolic pathways in the liver and that Sirt2 exhibited most of its regulatory influence on proteins within the cytosolic and nuclear compartments. Our functional studies showed that *Sirt2−/−* male mice had reduced gluconeogenic capacity and mitochondrial function. The defective metabolic pathways in the liver resulted in systemic effects, i.e., *Sirt2−/−* males had reduced fatty liver content, reduced fat mass, and displayed mild hypoglycemia. Among the hyper-acetylated proteins in the cytosol of *Sirt2−/−* livers were four enzymes involved in glucose metabolism, including Ldha, which plays key roles in both glycolysis and gluconeogenesis by maintaining a balance of pyruvate and lactate that persists throughout the whole body. Indeed, Ldha enzymatic activity was reduced in the *Sirt2−/−* male liver. Ldha tetramerization analysis and further interrogation of lysine residues K126 and K243 (which were found to be hyper-acetylated with the absence of sirt2 in this study) could uncover evidence that Sirt2 regulates Ldha stability. Therefore, a better understanding of this apparent relationship between Sirt2 and Ldha, specifically, how Sirt2 affects Ldha acetylation and overall function, is an important future direction within this space.

Of note, Sirt2 ablation appeared to specifically affect males compared to females, adding to the current body of evidence suggesting that metabolic processes are subject to sexual dimorphism [31,32]. With our unbiased proteomic approach, we found that *Sirt2−/−* male livers had increased overall protein acetylation levels, but not nearly as high as the acetylation levels in females. Despite data from males that suggested Sirt2 actively deacetylated several proteins, the accumulation of acetyl groups on lysine residues of hepatic metabolic pathways was clearly tolerated in female mice. Furthermore, the whole-body changes observed in *Sirt2−/−* mice might not necessarily be a detriment to the animal. Rather, decreased adipose tissue, lower liver triglycerides, and lower glucose levels could be viewed as beneficial in the context of metabolic diseases such as type 2 diabetes and obesity. A previous study on Sirt2 knockout male mice found significantly greater fat mass after 12 weeks on a 60% fat diet [33]. However, they also included a cohort of mice on standard low-fat chow (which we used throughout our studies) and observed that knockout males had ~10% more body fat and ~15% more lean mass. Thus, knockout males had lower adiposity when expressed as percent body fat, which agrees with our results. Further work is needed to assess why knockout males would be leaner on a low-fat diet but more obese on an ultra-high-fat diet.

Further work will also be necessary to elucidate Sirt2’s influence on mitochondrial biology. It is possible that changes in mitochondrial function, i.e., supply of ATP, could be contributing to the defect in gluconeogenesis. In the male liver, we observed significantly increased acetylation of numerous mitochondrial proteins in the absence of Sirt2, and mitochondrial respiration was significantly suppressed. In our mass spectrometry analysis, Cox5b, a subunit of Complex IV, was reduced by ~40% in male Sirt2−/− liver, but no other respiratory chain complexes showed altered expression in males (Supplemental Table S4). In female Sirt2−/− mice, two subunits of Complex I were reduced to a similar degree (Supplemental Table S5). Future work is needed to interrogate whether these changes play a role in respiratory chain function in Sirt2−/− mice. The electron transport chain proteins were not deregulated at the transcript level, as detected by RNAseq. It is possible that the decreases are due to the loss of Sirt2 action within the mitochondria, i.e., by acetylation changes affecting protein stability or turnover. Yet, we found no evidence of Sirt2 localizing to the mitochondria in wild type mouse liver. This was in contrast to an earlier report by Liu et al. that Sirt2 localizes to the mitochondria in mouse brain tissue and mouse embryonic fibroblasts [34]. It is possible that Sirt2 localizes to different places in different organs. Another difference between our work and that of Liu et al. is that we used gradient density centrifugation to purify mitochondria away from the endoplasmic reticulum, nuclei, and other organelles. Based on our results, we postulate that Sirt2 influences mitochondria from outside the organelle, a conclusion further supported by the work of others [12,35,36]. Sirt2 may influence mitochondrial dynamics, intracellular NAD+ balance, substrate supply to the mitochondria, or a combination of these. Intriguingly, in Sirt2-/- liver we observed significant hyperacetylation on four mitochondrial carrier proteins−Slc25a1, Slc25a5, Slc25a13, and Slc25a42−which encode membrane-spanning exchangers for citrate/malate, ATP/ADP, aspartate/glutamate, and CoA/ADP, respectively. Three of these four proteins (all except Slc25a42) also co-immunoprecipitated with tagged Sirt2 in our pulldown experiment. We speculate that Sirt2 may interact with these membrane-spanning proteins from the inner membrane space, possibly thereby regulating the transport of critical metabolic intermediates. Again, further work will be necessary to further interrogate the role of Sirt2 in mediating intercompartment metabolite transport and homeostasis.

## Figures and Tables

**Table 1:** Reactome pathway analysis of proteins showing hyperacetylation upon Sirt2 ablation.

Pathway Name	Entities *p*-Value
Metabolism of amino acids and derivatives	1.11 × 10^−16^
Metabolism	1.11 × 10^−16^
Eukaryotic Translation Elongation	5.82 × 10^−11^
The citric acid (TCA) cycle and respiratory electron transport	2.25 × 10^−10^
Selenoamino acid metabolism	2.51 × 10^−10^
Translation initiation complex formation	4.83 × 10^−10^
Ribosomal scanning and start codon recognition	4.83 × 10^−10^
Activation of the mRNA upon binding of the cap-binding complex and eIFs	5.98 × 10^−10^
SARS-CoV-1 modulates host translation machinery	6.42 × 10^−10^
L13a-mediated translational silencing of Ceruloplasmin expression	7.73 × 10^−10^
GTP hydrolysis and joining of the 60S ribosomal subunit	8.88 × 10^−10^
Formation of the ternary complex, and subsequently, the 43S complex	1.09 × 10^−9^
Response of EIF2AK4 (GCN) to amino acid deficiency	1.35 × 10^−9^
Peptide chain elongation	1.69 × 10^−9^
Protein localization	2.02 × 10^−9^

**Table 2:** Top 10 Hyperacetylated Sites on Nuclear Proteins.

Protein	Name	Compartment(s)	Site(s)	Log2FC
*Hint1*	Histidine triad nucleotide-binding protein 1	Nuc, Cyto	K21	14.7
Myc	Myc proto-oncogene	Nuc, Cyto	K149	12.7
Rps2	40S ribosomal protein S2	Nuc, Cyto	K275	11.2
Erh	Enhancer of rudimentary homolog	Nuc	K12	5.6
Rela	Transcription factor p65	Nuc, Cyto	K122	4.7
CK054	Ester hydrolase C11 or f54 homolog	Nuc	K144	4.6
Hmgb1	High mobility group protein B1	Nuc, Cyto	K12	4.4
Rundc1	RUN domain-containing protein 1	Nuc, Cyto	K596	3.7
Lmnb2	Lamin-B2	Nuc	K381	3.5
Hist1h4a	Histone H4	Nuc	K92	3.2

## Data Availability

The original contributions presented in the study are included in the Supplementary Materials; further inquiries can be directed to the corresponding author. Raw data and complete MS data sets have been uploaded to the Center for Computational Mass Spectrometry, to the MassIVE repository at UCSD, and can be downloaded using the following FTP link: ftp://MSV000095391@massive.ucsd.edu or via the MassIVE website: https://massive.ucsd.edu/ProteoSAFe/dataset.jsp?task=2b053f951e0947eba7bbd4049ae91faf (MassIVE ID number: MSV000095391; ProteomeXchange ID: PXD054074). [**Note to the reviewers:** To access the data repository MassIVE (UCSD) for MS data, please use: Username: MSV000095391_reviewer; Password: winter].

## Supplementary Materials

The following supporting information can be downloaded at https://www.mdpi.com/article/10.3390/biom14091160/s1: Table S1: Acetylated peptides: Sirt2-KO vs. WT for male mice; Table S2: Acetylated peptides: Sirt2-KO vs. WT for female mice; Table S3: RNA-seq significant mRNAs; Table S4: Protein levels, T2KO versus WT male; Table S5: Protein groups: Sirt2-KO vs. WT for female mice.
