# Supplementary material for: Sirt2 Regulates Liver Metabolism in a Sex-Specific Manner
Source: Biomolecules. 2024 Sep 15;14(9):1160. doi: 10.3390/biom14091160 (PMC11430619; doi:10.3390/biom14091160)
Supplement: Supplementary file 1 [file biomolecules-14-01160-s001.zip › biomolecules-3185584 supplementary.pdf]

Uncropped Gel Images

Schmidt et al

Figure 1f.

Anti-Sirt2 immunoblot.

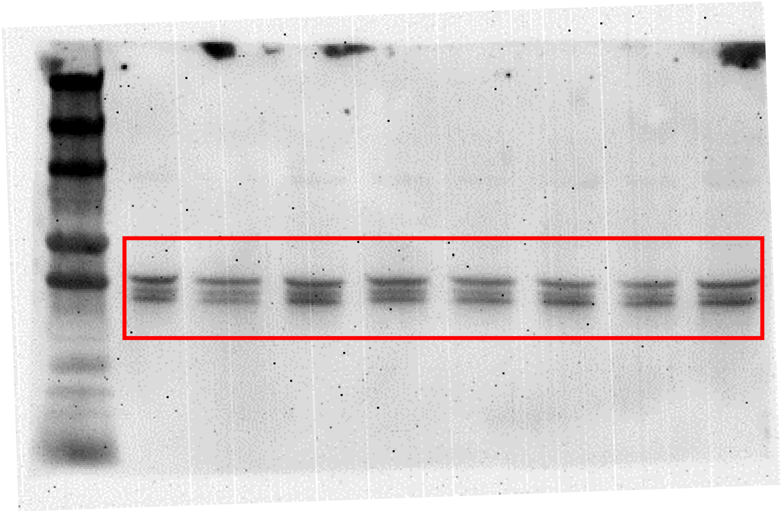

Anti-Actin loading control

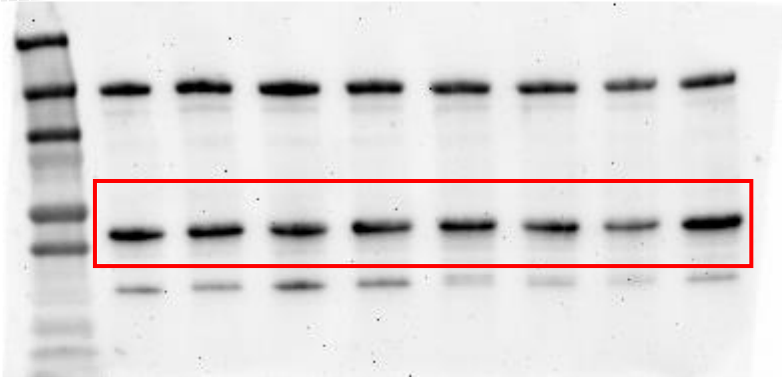

Figure 2 d

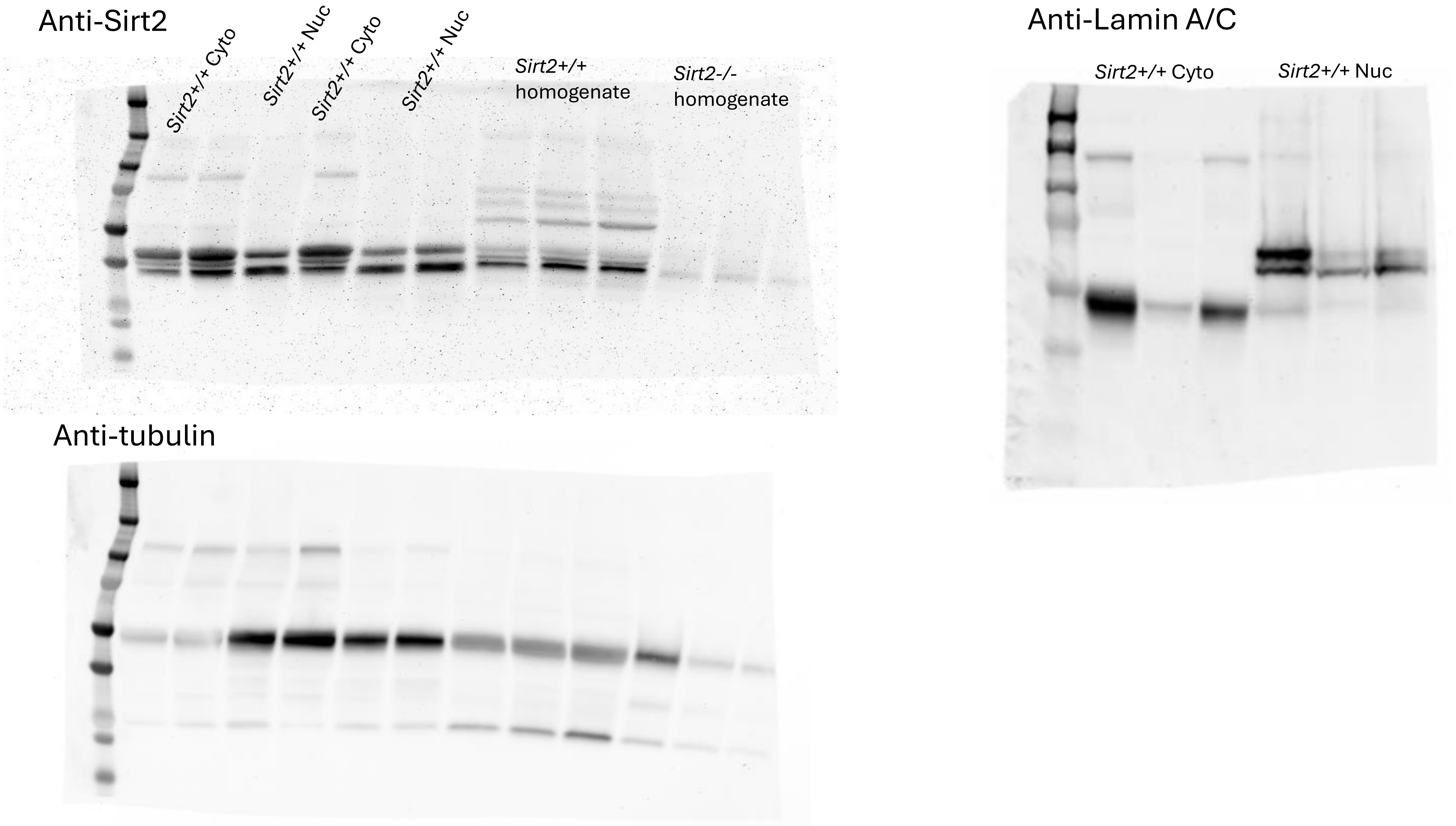

*Stripped, re-probed, and exposed from original (above)

Anti-Sirt2

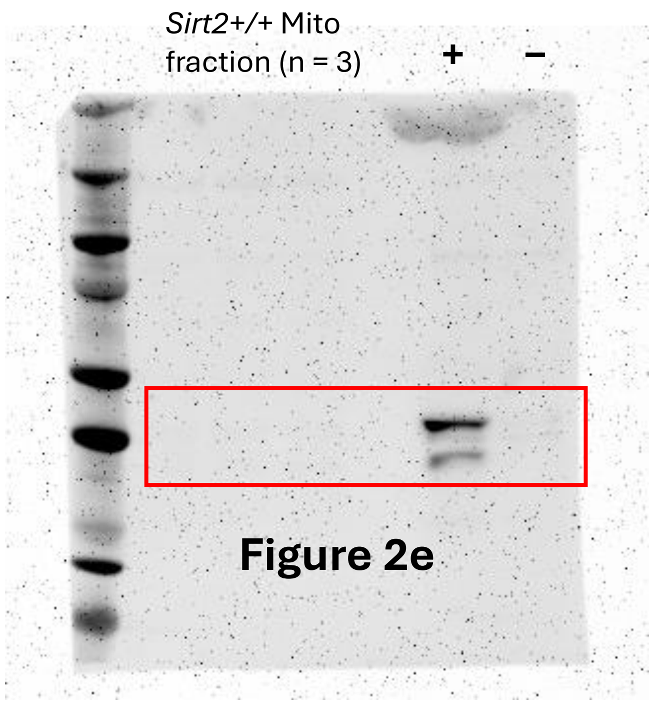

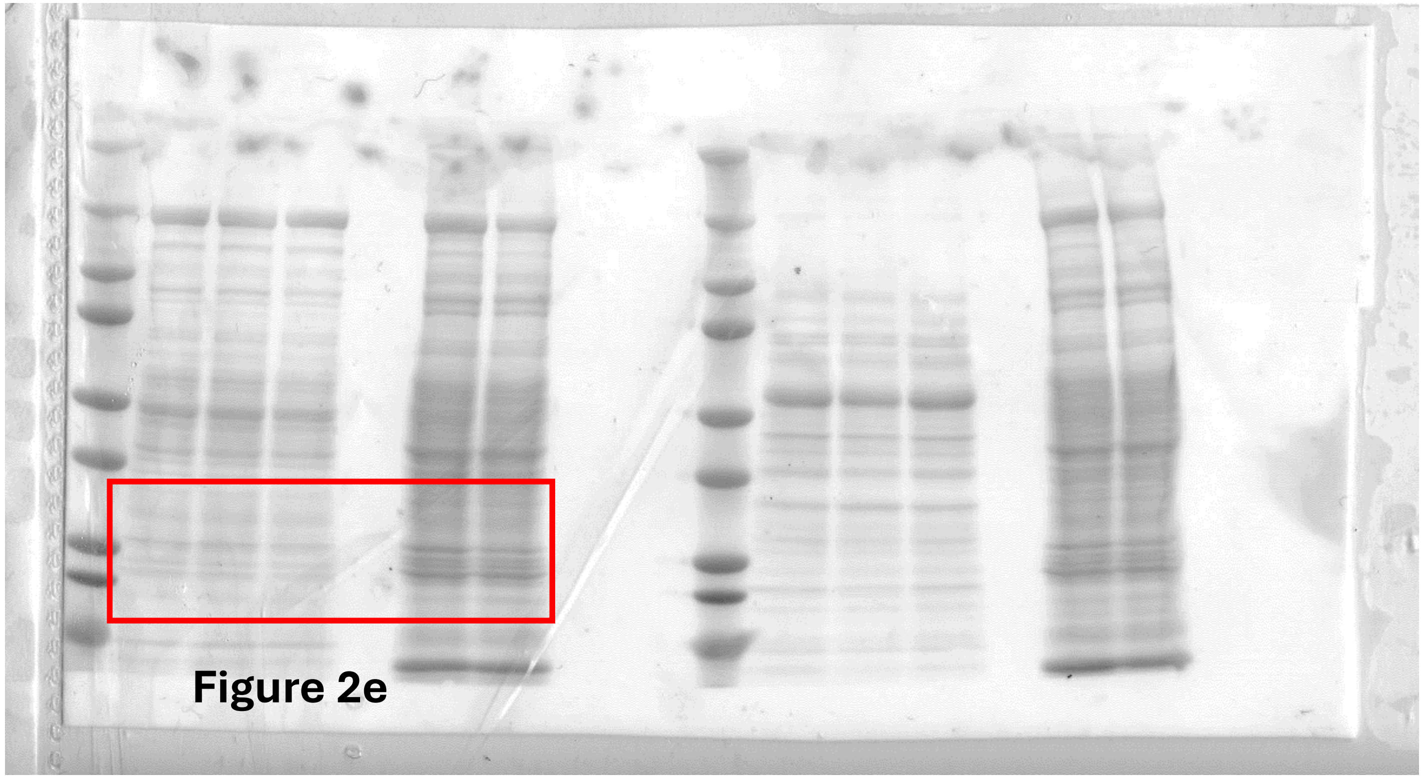

Mito fraction ponceau loading control

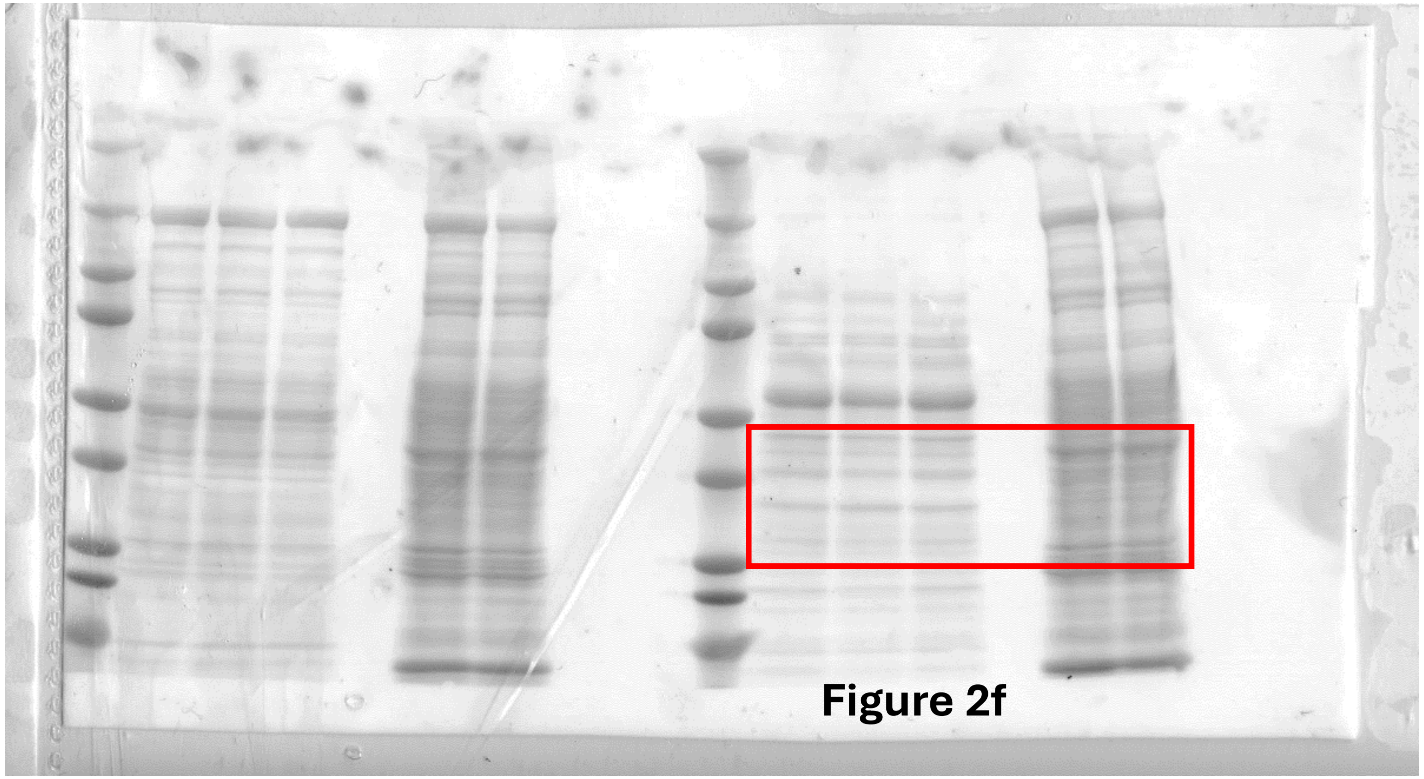

Peroxisome fraction ponceau loading control

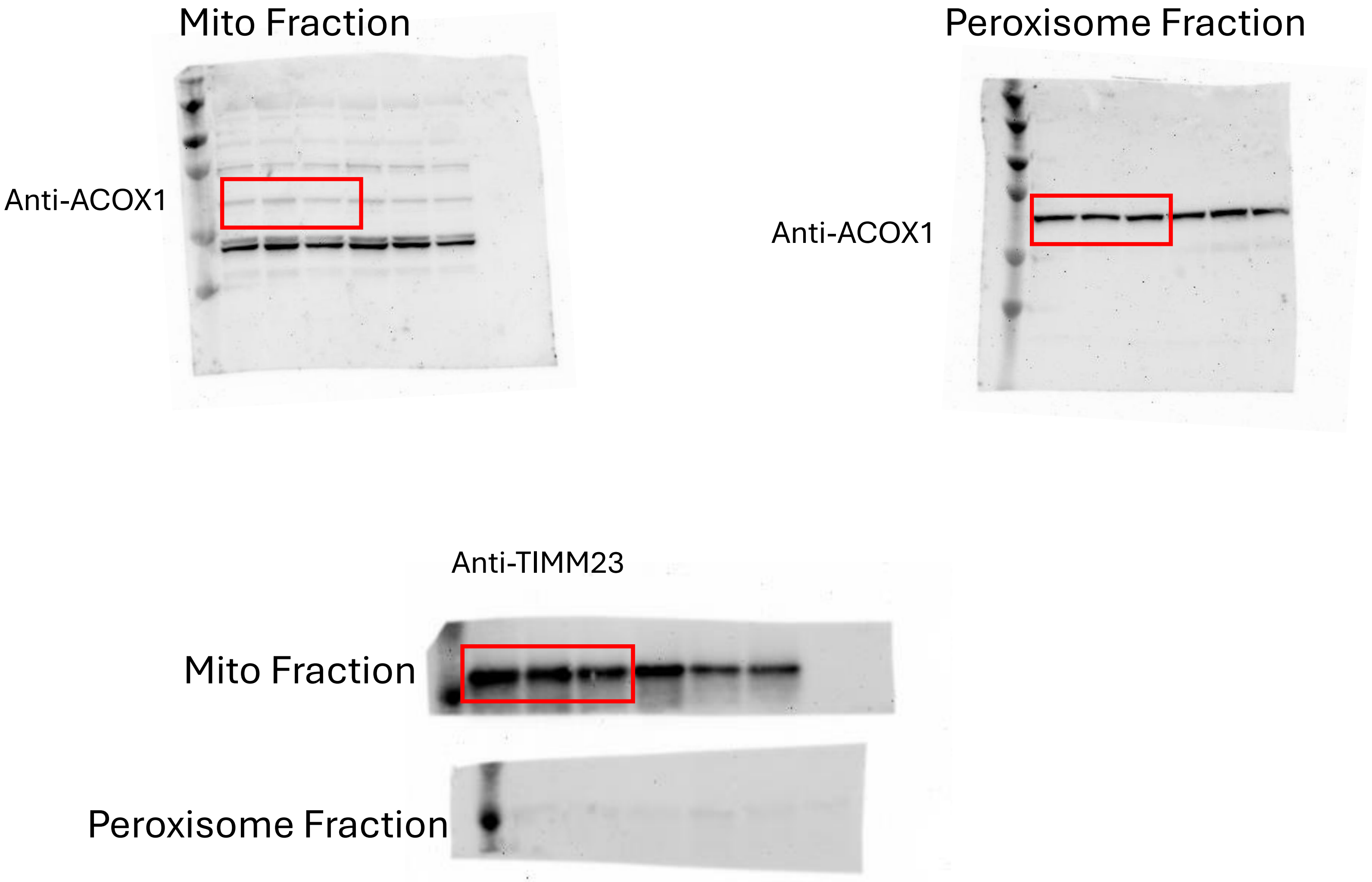

* Stripped, re-probed, and cut from original membranes (above). Exposed at the same time.
