# Supplementary material for: Sirt2 Regulates Liver Metabolism in a Sex-Specific Manner
Source: Biomolecules. 2024 Sep 15;14(9):1160. doi: 10.3390/biom14091160 (PMC11430619; doi:10.3390/biom14091160)
Supplement: Supplementary file 1 [file biomolecules-14-01160-s001.zip › Full membrane figs_sirt2 metabolism.pptx]

## Slide 1
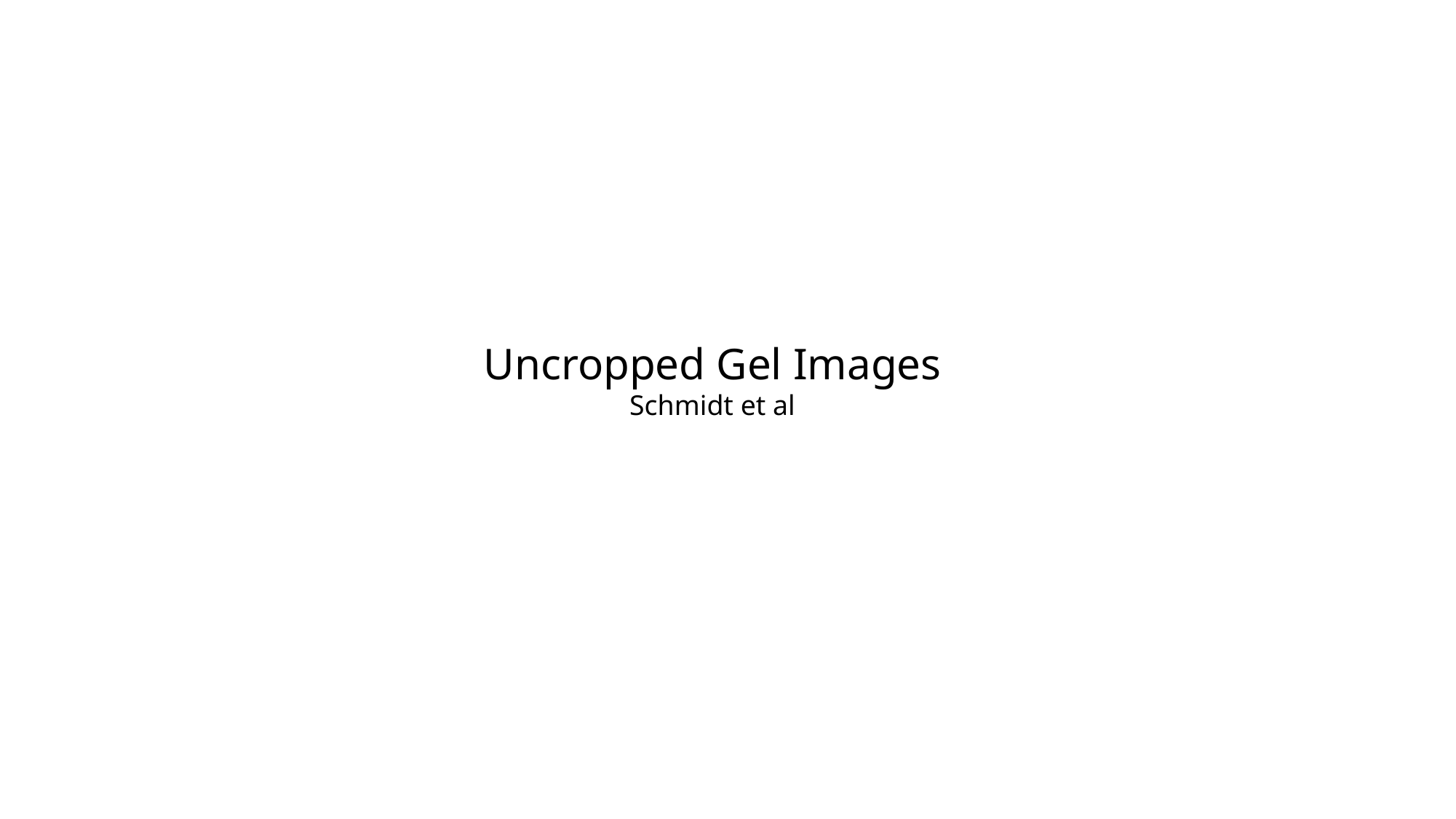

Uncropped Gel Images
Schmidt et al

## Slide 2
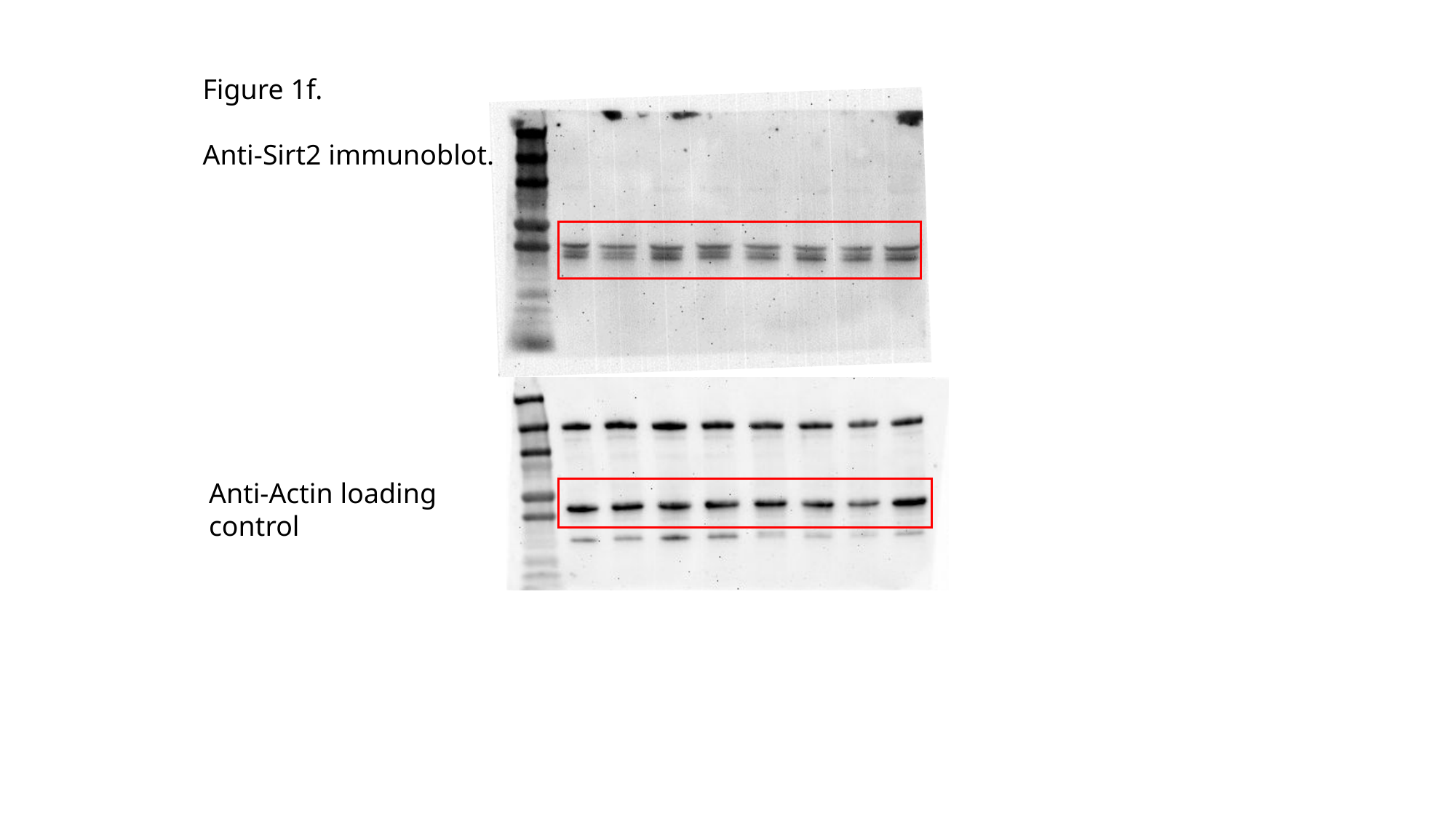

Figure 1f.
Anti-Sirt2 immunoblot.
Anti-Actin loading control

## Slide 3
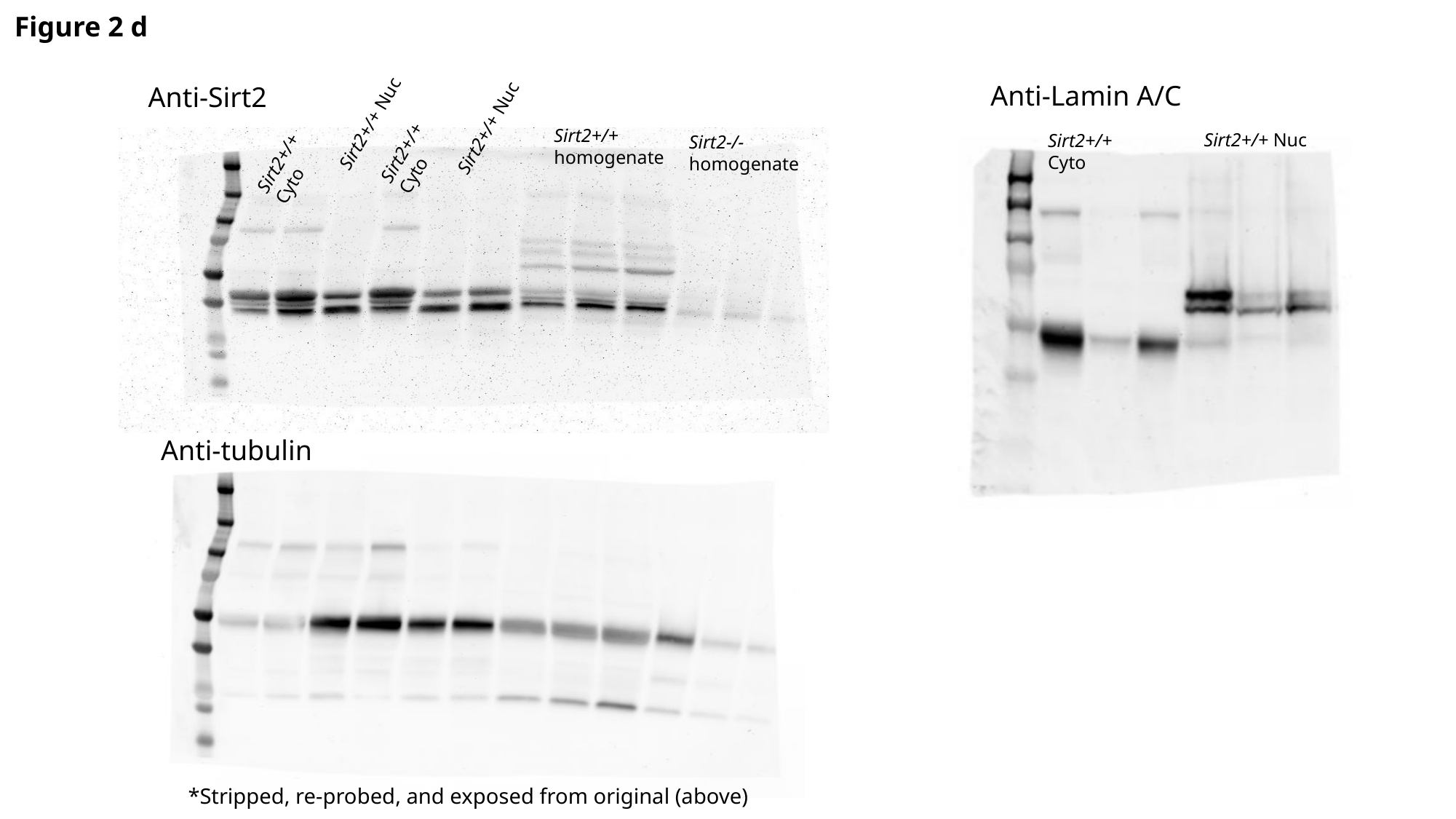

Figure 2 d
Anti-Lamin A/C
Anti-Sirt2
Sirt2+/+ Nuc
Sirt2+/+ Nuc
Sirt2+/+ homogenate
Sirt2+/+ Cyto
Sirt2+/+ Nuc
Sirt2+/+ Cyto
Sirt2-/- homogenate
Sirt2+/+ Cyto
Anti-tubulin
*Stripped, re-probed, and exposed from original (above)

## Slide 4
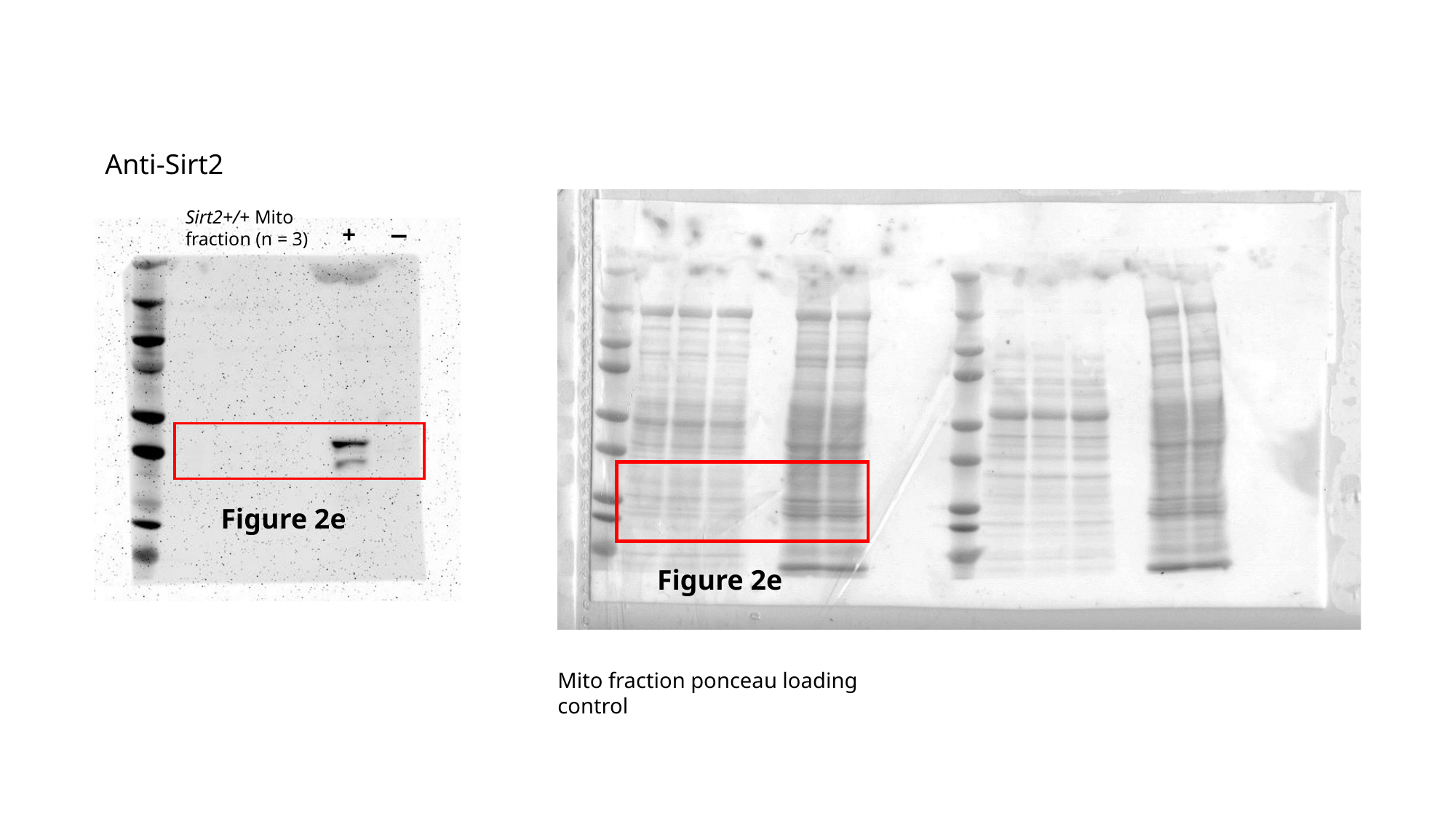

Anti-Sirt2
Sirt2+/+ Mito fraction (n = 3)
+
−
Figure 2e
Figure 2e
Mito fraction ponceau loading control

## Slide 5
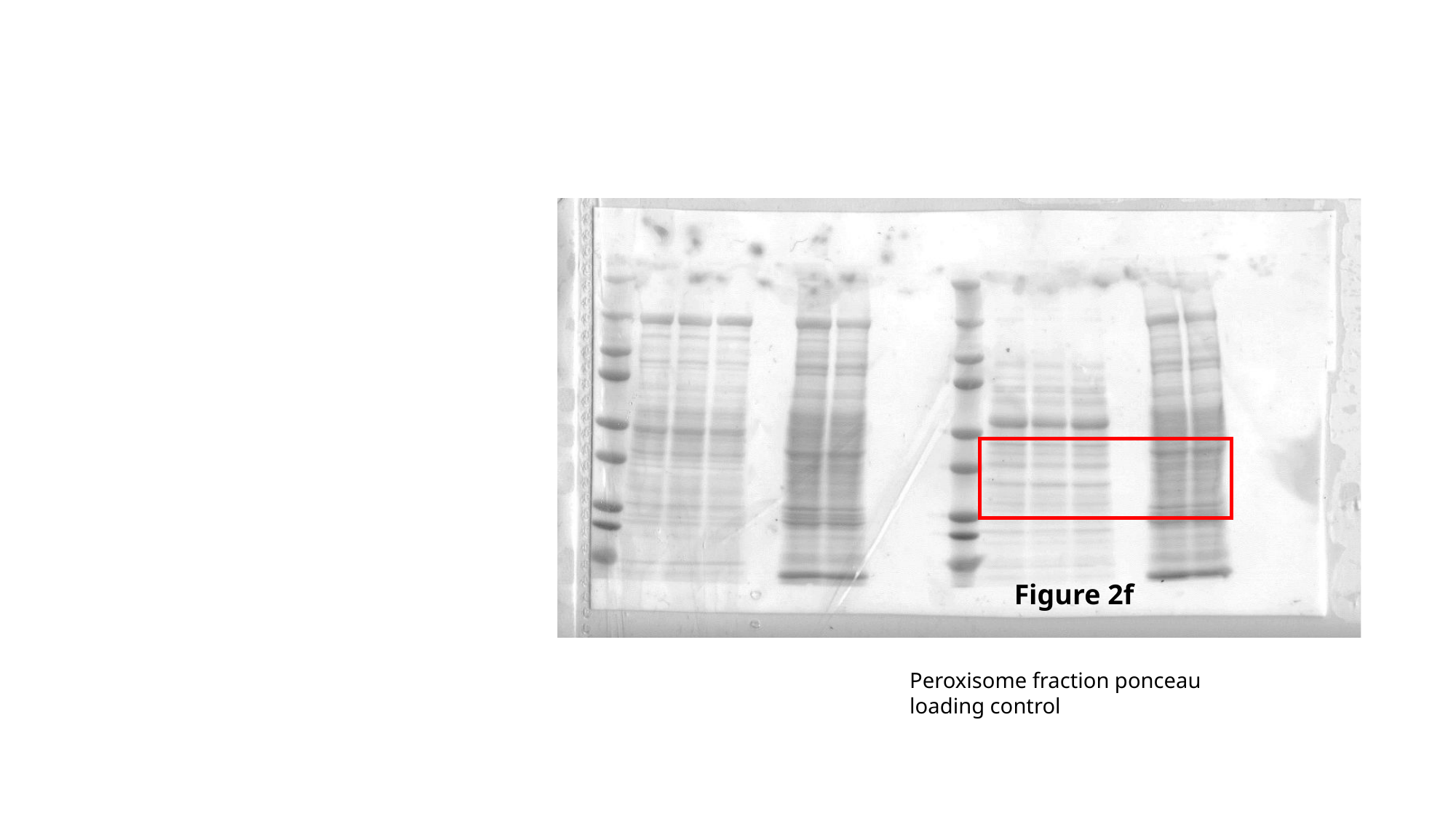

Figure 2f
Peroxisome fraction ponceau loading control

## Slide 6
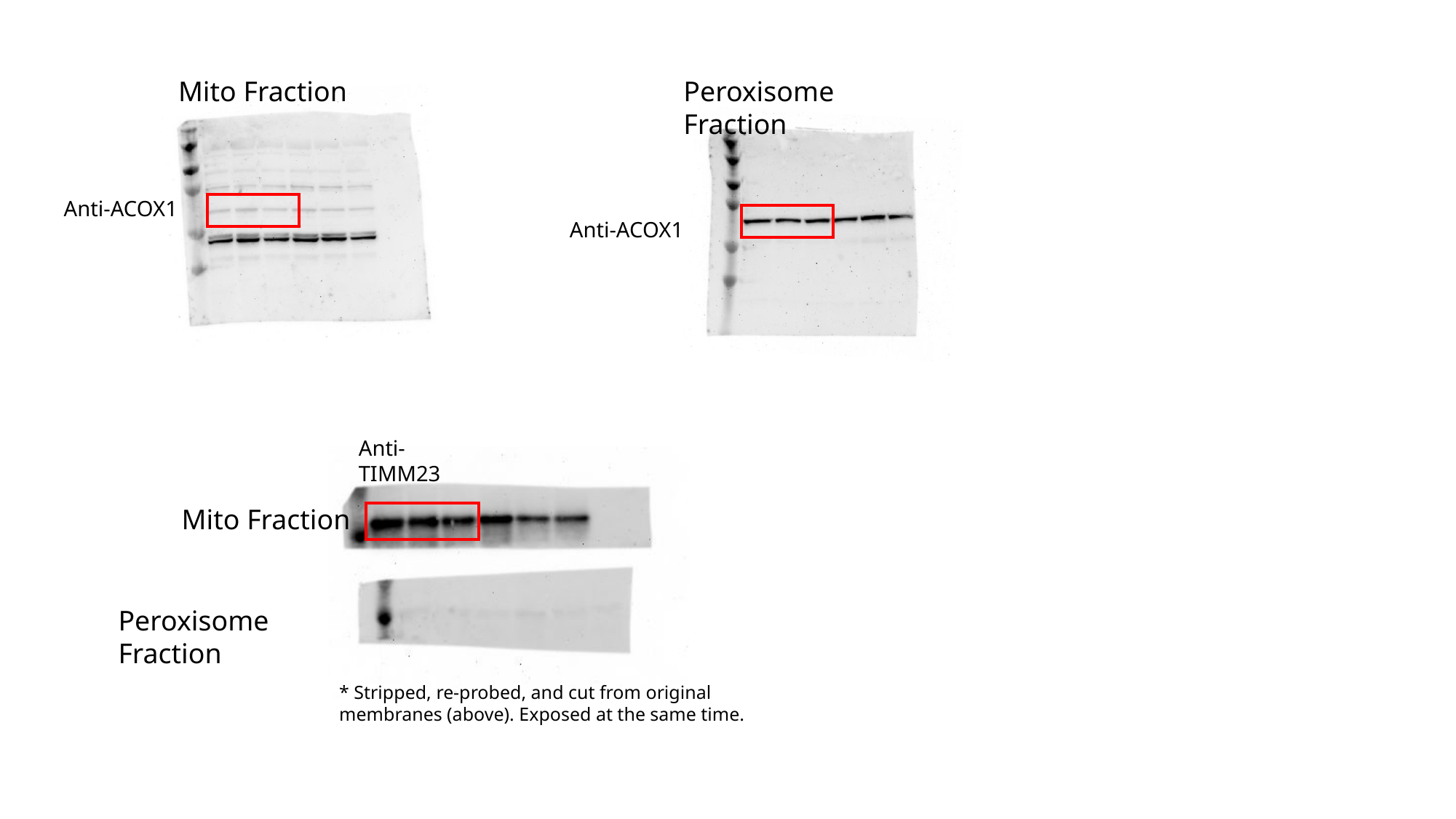

Mito Fraction
Peroxisome Fraction
Anti-ACOX1
Anti-ACOX1
Anti-TIMM23
Mito Fraction
Peroxisome Fraction
* Stripped, re-probed, and cut from original membranes (above). Exposed at the same time.
