# Supplementary material for: Sirt2 Regulates Liver Metabolism in a Sex-Specific Manner
Source: Biomolecules. 2024 Sep 15;14(9):1160. doi: 10.3390/biom14091160 (PMC11430619; doi:10.3390/biom14091160)
Supplement: Supplementary file 1 [file biomolecules-14-01160-s001.zip › Supplemental Information_Schmidt et al_Sirt2 regulates liver metabolism in a sex dependent manner.pdf]

**Supplemental Figures**

Sirt2 Regulates Liver Metabolism in a Sex-Dependent Manner

Alexandra V. Schmidt, et al

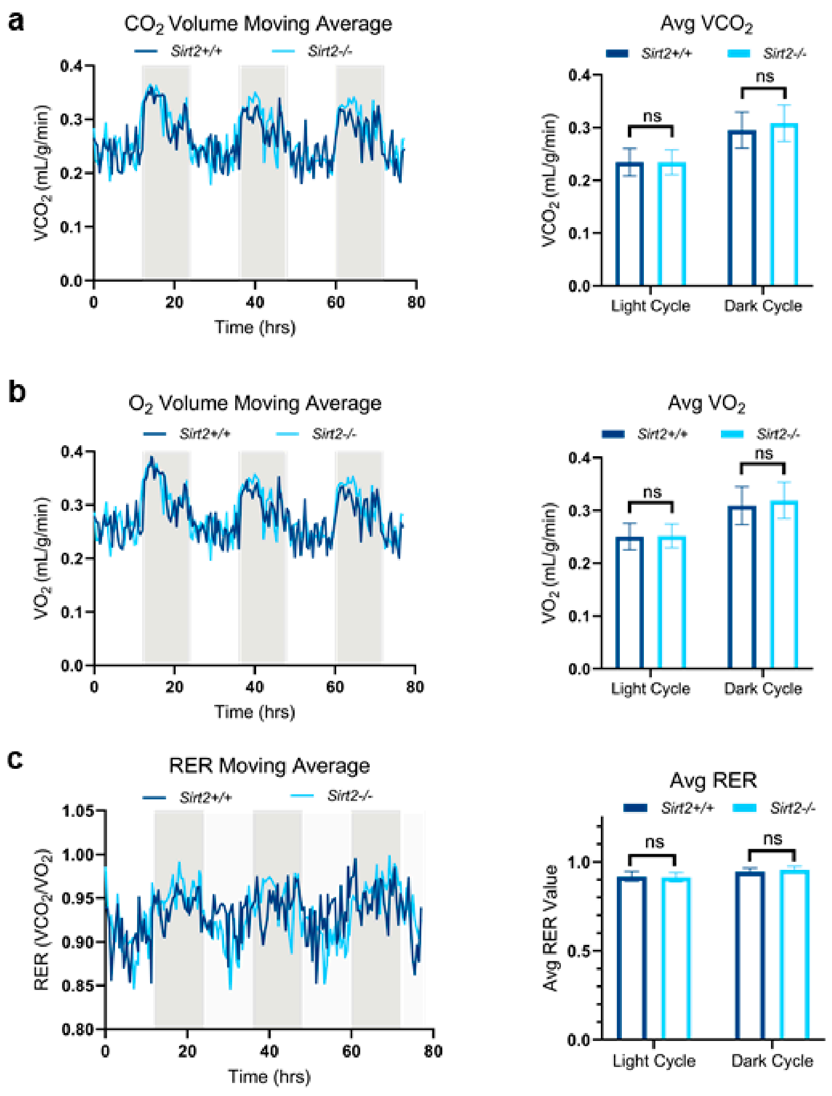


**Figure S1.** Indirect Calorimetry (IDC) was performed for 72 hrs in Sirt2-/- (N=3) vs Sirt2+/+ (N=4) males to measure whole-body respiration. IDC is a reflection of FAO/glycolytic flux in major metabolic tissues; such as, liver, brain, muscle, and kidneys. **(a)** There was no difference in $CO_2$ generation during the light or dark cycle. **(b)** There was no difference between *Sirt2-/-* and *Sirt2+/+* mice in $O_2$ uptake. **(c)** There was no difference in RER in *Sirt2-/-* vs *Sirt2+/+* mice over the course of 72 hrs, indicating no significant differences in systemic flux. All comparisons made using Student's nonparametric t-tests.

**Supplemental Methods**

**Proteomics and Acetylomics Analysis by Data-Independent Acquisition**

1. **Sample preparation for liver acetylomics analysis**

Approximately 100 mg of mouse liver tissues were collected from 4 different conditions with 3-4 replicates each: i) wild-type male (WT_Male, n=4), ii) SIRT2(-/-) male (SIRT2-KO_Male, n=4), iii) wild-type female (WT_Female, n=3) and iv) SIRT2(-/-) female (SIRT2-KO_DC, n=3). Frozen livers were immersed in 450 μL of lysis buffer containing 8 M urea, 200 mM triethylammonium bicarbonate (TEAB), pH 8, 75 mM sodium chloride, 1 μM trichostatin A, 3 mM nicotinamide, and 1x protease/phosphatase inhibitor cocktail (Thermo Fisher Scientific, Waltham, MA), and homogenized for 2 cycles with a Bead Beater TissueLyser II (Qiagen, Germantown, MD) at 24 Hz for 3 min each. Lysates were clarified by spinning at 15,700 x g for 15 min at 4°C, and the supernatant containing the soluble proteins was collected. Protein concentrations were determined using a Bicinchoninic Acid Protein (BCA) Assay (Thermo Fisher Scientific, Waltham, MA).

For acetylome analysis, 5.1 mg of protein from each sample were aliquoted and samples were brought to an equal volume using a solution of 8 M urea in 50 mM TEAB, pH 8. Proteins were reduced using 20 mM dithiothreitol (DTT) in 50 mM TEAB for 30 min at 37 °C, and after cooling to room temperature, alkylated with 40 mM iodoacetamide (IAA) for 30 min at room temperature in the dark. Samples were diluted 4-fold with 50 mM TEAB, pH 8, and proteins were digested overnight with a solution of sequencing-grade trypsin (Promega, San Luis Obispo, CA) in 50 mM TEAB at a 1:50 (wt:wt) enzyme:protein ratio at 37°C. This reaction was quenched with 1% formic acid (FA) and the sample was clarified by centrifugation at 2,000 x g for 10 min at room temperature. Clarified peptide samples were desalted with Oasis 30-mg Sorbent Cartridges (Waters, Milford, MA). The digests were re-suspended in 1.4 mL of immunoaffinity purification (IAP) buffer (Cell Signaling Technology, Danvers, MA) containing 50 mM 4-morpholinepropanesulfonic acid (MOPS)/sodium hydroxide, pH 7.2, 10 mM disodium phosphate, and 50 mM sodium chloride for PTM enrichment. Peptides were enriched for acetylation with anti-acetyl antibody conjugated to agarose beads from the Acetyl-Lysine Motif Kit (Cell Signaling Technology, Danvers, MA). This process was performed according to the manufacturer protocol, however each sample was incubated in half the recommended volume of washed beads. Peptides were eluted from the antibody-bead conjugates with 0.15% trifluoroacetic acid in water and were desalted using C18 stagetips made in-house. Samples were vacuum dried and re-suspended in 0.2% FA in water.

For proteome analysis, 100-ug aliquots of each sample were solubilized with 4% SDS and 50 mM TEAB, pH 8. Proteins were reduced with 20 mM DTT (10 min at 50 °C followed by 10 min at room temperature) and then alkylated with 40 mM IAA (30 min at room temperature in the dark). Samples were acidified with a final concentration of 1.2% phosphoric acid, and diluted with seven volumes S-trap buffer (90% methanol in 100 mM TEAB, pH 8). Samples were then loaded onto the S-trap mini spin columns (Protifi) and spun at 4,000 x g for 10 seconds. The S-Trap columns were washed with S-Trap buffer twice at 4,000 x g for 10 seconds each. A solution of sequencing grade trypsin in 50 mM TEAB at a 1:25 (w/w) enzyme:protein ratio was then added, and after a 1-hour incubation at 47°C, trypsin solution was added again at the same ratio and proteins were digested overnight at 37°C. Peptides were sequentially eluted with 50 mM TEAB (spinning at 1,000 x g for 1 min), 0.5% FA in water (spinning at 1,000 x g for 1 min), and 50% ACN in 0.5% FA (spinning at 4,000 x g for 1 min). After vacuum drying, samples were resuspended in 0.2% FA in water

and desalted with Oasis 10-mg Sorbent Cartridges (Waters). All samples were vacuum dried and resuspended in 0.2% FA in water at a final concentration of 1 µg/µL.

Finally, indexed retention time standard peptides (iRT; Biognosys) were spiked in all samples according to manufacturer's instructions.

## 2. Mass spectrometric analysis

LC-MS/MS analyses were performed on a Dionex UltiMate 3000 system online coupled to an Orbitrap Eclipse Tribrid mass spectrometer (Thermo Fisher Scientific, San Jose, CA). The solvent system consisted of 2% ACN, 0.1% FA in water (solvent A) and 98% ACN, 0.1% FA in water (solvent B). Proteolytic peptides were loaded onto an Acclaim PepMap 100 C18 trap column with a size of 75 µm x 20 mm and 3 µm particle size (Thermo Fisher Scientific) for 10 min at 5 µL/min with 100% solvent A. For the protein lysates (protein level analysis), an amount of 200 ng was loaded, and for the enriched acetylated peptides (PTM level analysis), 4 µL of each PTM-enriched sample were injected. Peptides were eluted on to an Acclaim PepMap 100 C18 analytical column sized as follows: 75 µm x 50 cm, 3 µm particle size (Thermo Fisher Scientific) at 0.3 µL/min using the following gradient of solvent B: 2% for 10 min, linear from 2% to 20% in 125 min, linear from 20% to 32% in 40 min, up to 80% in 1 min, 80% for 9 min, and down to 2% in 1 min. The column was equilibrated with 2% of solvent B for 29 min (total gradient length = 215 min).

All samples were acquired in data-independent acquisition (DIA) mode. Full MS spectra were collected at 120,000 resolution (AGC target: 3e6 ions, maximum injection time: 60 ms, 350-1,650 m/z), and MS2 spectra at 30,000 resolution (AGC target: 3e6 ions, maximum injection time: Auto, NCE: 27, fixed first mass 200 m/z). The DIA precursor ion isolation scheme consisted of 26 variable windows covering the 350-1,650 m/z mass range with an overlap of 1 m/z.

## 3. DIA data processing and statistical analysis

DIA data was processed in Spectronaut (version 16.0.220606.53000) using directDIA for both the protein level as well as PTM-enriched samples. Data was searched against the Mus musculus proteome with 58,430 protein entries (UniProtKB-TrEMBL), accessed on 01/31/2018. Trypsin/P was set as digestion enzyme and two missed cleavages were allowed. Cysteine carbamidomethylation was set as fixed modification, and methionine oxidation and protein N-terminus acetylation as variable modifications. Data extraction parameters were set as dynamic and non-linear iRT calibration with precision iRT was selected. Identification was performed using 1% precursor and protein q-value. For the protein lysate samples, quantification was based on the extracted ion chromatograms (XICs) of 3 – 6 MS2 fragment ions, and local normalization was applied. iRT profiling was selected. For the PTM-enriched samples, lysine acetylation was additionally set as variable modification. PTM localization was selected with a probability cutoff of 0.75. Quantification was based on the XICs of 3 – 6 MS2 fragment ions, specifically b- and y-ions, without normalization as well as data filtering using q-value sparse. Grouping and quantitation of PTM peptides were accomplished using the following criteria: minor grouping by modified sequence and minor group quantity by mean precursor quantity.

Differential expression analysis was performed using a paired t-test and p-values were corrected for multiple testing, specifically applying group wise testing corrections using the Storey method. For whole lysate (protein level) analysis, protein groups are required with at least two unique peptides. For determining differential protein changes, a p-value < 0.01 and absolute Log2(fold-change) > 0.58 are required to qualify as 'significantly-altered'. For the PTM analysis, each acetylated peptide is quantified individually comparing conditions with significance cutoffs of p-value < 0.01 and absolute Log2(fold-change) > 0.58.
